# Anti–SARS-CoV-2 Natural Products as Potentially Therapeutic Agents

**DOI:** 10.3389/fphar.2021.590509

**Published:** 2021-05-27

**Authors:** Cheorl-Ho Kim

**Affiliations:** Molecular and Cellular Glycobiology Unit, Department of Biological Sciences, Sungkyunkhwan University, Suwon, South Korea

**Keywords:** SARS-CoV-2, ACE2 inhibitor, natural products, replication inhibitor, virus entry blocker

## Abstract

Severe acute respiratory syndrome–related coronavirus-2 (SARS-CoV-2), a β-coronavirus, is the cause of the recently emerged pandemic and worldwide outbreak of respiratory disease. Researchers exchange information on COVID-19 to enable collaborative searches. Although there is as yet no effective antiviral agent, like tamiflu against influenza, to block SARS-CoV-2 infection to its host cells, various candidates to mitigate or treat the disease are currently being investigated. Several drugs are being screened for the ability to block virus entry on cell surfaces and/or block intracellular replication in host cells. Vaccine development is being pursued, invoking a better elucidation of the life cycle of the virus. SARS-CoV-2 recognizes O-acetylated neuraminic acids and also several membrane proteins, such as ACE2, as the result of evolutionary switches of O-Ac SA recognition specificities. To provide information related to the current development of possible anti–SARS-COV-2 viral agents, the current review deals with the known inhibitory compounds with low molecular weight. The molecules are mainly derived from natural products of plant sources by screening or chemical synthesis via molecular simulations. Artificial intelligence–based computational simulation for drug designation and large-scale inhibitor screening have recently been performed. Structure–activity relationship of the anti–SARS-CoV-2 natural compounds is discussed.

## Introduction

### General Virology of Coronaviruses

The coronaviruses (CoVs) target humans and animals with exchangeable infectivity, causing a zoonotic outbreak. SARS-CoV-2 or 2019-nCoV spreads and causes the human life crisis of COVID-19 by infecting the human respiratory tract and causing pneumonia (Zhou et al., 2020). In addition, SARS-CoV-2 spreads by easy transmission among people, and COVID-19 patients exhibit flu-like symptoms such as fever and cough. Enveloped CoVs contain positive ssRNA genomes with relatively small RNAs (approximately 30 kb). They are classified into the *Riboviria*–*Nidovirales*–*Cornidovirineae*–*Coronaviridae*–*Orthocoronavirinae*–CoV genus (α-, β-, γ-, and *δ*-CoV). Most mammals are infected by *α*-CoV and *β*-CoV only, while avians and some mammals are infected by *δ*-CoV and *γ*-CoV. SARS-CoV-2, belonging to the *β*-CoV genus, and bat SARS-like CoV-ZXC-21 are similar in their RNA genomes. The COVID-19–causing CoV isolates exhibit 79% identity with the previously named SARS-CoV and 50% identity with the Middle East respiratory syndrome (MERS) virus (Chan et al., 2020).

**FIGURE 1:**
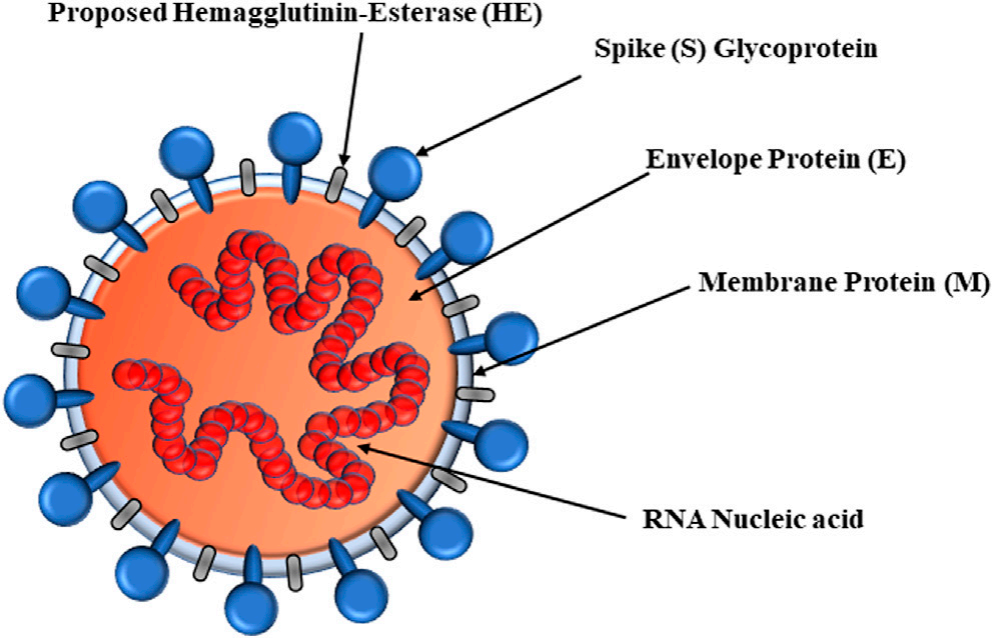
Structure of enveloped coronavirus virion.

SARS-CoV-2 viral proteins include RNA-dependent RNA polymerase (RdRp) and hemagglutinin-esterase (HE) enzymes as well as proteins including spike (S), envelope (E), membrane (M), and nucleocapsid (N) (Figure 1) (Tu et al., 2020). Nonstructural protein 3 (Nsp3), Nsp5, Nsp9, and Nsp12 RdRp are enzymes. The E-, S-, and M-proteins are embedded into the endoplasmic reticulum (ER) membrane and translocated to the ER–Golgi intermediate compartment (ERGIC). As the first step, the S-glycoprotein of SARS-CoV-2 binds to surfaced O-acetyl (Ac)-neuraminic acids of host cells. The neuraminic acid-O-Ac-esterase of HE evolved from the influenza C virus, nidoviruses, and salmon anemia virus (teleost orthomyxovirus). Fusion of S-glycoprotein and HE is important for CoV attachment to neuraminic acid–bearing host receptors (Tortorici et al., 2019). Therefore, the HE found in the β-CoV genus mediates viral attachment to O-Ac-neuraminic acids. The glycoproteins of the HA, HE, S, and HA-esterase-fusion protein (HEF) bind to the host receptor. However, certain α-CoV and γ-CoV are deficient of the neuraminic acid-O-Ac-esterases but bind to Ac-neuraminic acids or glycolyl-neuraminic acids. Murine CoVs esterize the C4-O-Ac (Smits et al., 2005). The SARS-CoV-2 S-glycoprotein N-terminal domain recognizes the surface entry site, binding to 9-O-Ac-neuraminic acid in a similar manner to CoV HEs as well as influenza C and D HEFs. Bovine CoV (BCoV) and human CoV (HCoV)-OC43 can recognize the 5,9-Ac2-neuraminic acids (Vlasak et al., 1988) and bear neuraminic acid 9-O-Ac-esterase. Most *β*-CoVs bind to the 9-O-Ac-neuraminic acids, but mutant strains target 4-O-Ac-neuraminic acids. Specifically, the HEs of *β*-CoVs recognize the 9-O-Ac-neuraminic acids, although certain species bind to the 4-O-Ac forms (Kim, 2020). In fact, 9-O-Ac-neuraminic acid is the recognition site for HCoV-OC43, β1-CoV, and SARS-CoV-2 S-glycoproteins, but MERS-CoV recognizes the α2,3-linkage. The CoV glycoproteins, BCoV HEs, and influenza virus HEFs are specific for 9-O-Ac-neuraminic acid (Mani et al., 2020; McKee et al., 2020), but the influenza HA is specific for glycolylneuraminic acids (Vogel et al., 2006). Upon interaction with neuraminic acid, host furin proteases cleave the S-glycoprotein to potentiate entry into host cells (Oliveira et al., 2017).

The current global COVID-19 pandemic is threatening the daily lives of human beings. The disease biology is a topic of interest. To overcome the disease, the academic society urgently needs to exchange the pandemic CoV-controlling drugs, but no truly effective agent has yet been discovered. In this review, antiviral candidate agents and the availability of natural compounds are discussed.

## Natural Products to Target and Inhibit Infection of Coronaviruses

Recently, natural phytochemicals that exhibit anti-CoV activity have been extensively summarized (Li et al., 2005). LMW molecules exhibit antiviral activity. Recently, development of anti-CoV drugs has also been applied for molecular docking via simulation approaches. Computer-based artificial intelligence technology contributes to the development of anti-CoV agents. Human angiotensin-converting enzyme (ACE)-2, papain-like protease (PLpro), main 3C-like protease (3CLpro), RdRp, helicase, N7 methyltransferase, human DDP4, receptor-binding domain (RBD), cathepsin L, type II transmembrane (TM) Ser-protease, or transmembrane protease serine (TMPRSS)-2 is mainly targeted. CoV 3CLpro and PLpro are polyprotein-specific viral proteases. RdRp is a complementary RNA strand synthetic replicase. Remdesivir inhibits RdRp in the ssRNA genome of CoV, where the RdRp mediates RNA replication and remdesivir acts as an ATP analog and thus inhibits RdRp.

Currently, effective anti-CoV agents are not available, although several drugs have been prescribed and some natural compounds exhibit antiviral activity. Natural resources are a tremendous treasure trove of chemical compounds that are applicable for various viral infections. Natural products are produced by the metabolic pathways of a given organism, but humans utilize them for their benefits from the modern view of pharmacology. Therefore, phytochemicals have been screened to test their effectiveness against viruses, and some natural products inhibit the infection and amplification of viruses with a broad antiviral spectrum (Pour et al., 2019). Naturally occurring compounds such as artemisinin, baicalin, curcumin, rutin, glycyrrhizic acid, hesperidin, hesperetin, and quercetin have been examined for their anti-CoV activity by various assay based on the viral life cycle. However, none of the natural compounds have direct antiviral activity against CoVs or other RNA viruses. Only GA has been frequently described to be the most active component in several previous articles. Indeed, the molecular action mechanisms of the natural products are not specific because current candidates of natural antiviral agents are mainly examined by using *in vitro* cell-based assays or computer modeling through docking simulation before application to animal and clinical studies (Li et al., 2005; Packer and Cadenas, 2011). Conventional approaches to natural products were a mix of chemical analysis and structure–function relationship analysis. Recent AI-aided approaches combined with *in silico* computational simulation is cost-effective for the prediction of chemical compounds (Müller et al., 2018). Currently, new concepts of AI-aided *in silico* computational approaches have evolved for drug prediction based on drug candidate–ligand/receptor interaction. These approaches utilize known structures of the molecules to predict *in silico* docking molecules. Fundamental limitations of these AI *in silico* approaches have also been identified. In fact, in blocking or inhibiting viral proteases, S-glycoprotein, and the entry of SARS-CoV-2, several plant compounds have been suggested through computer simulation techniques (Li et al., 2005).

**FIGURE 2:**
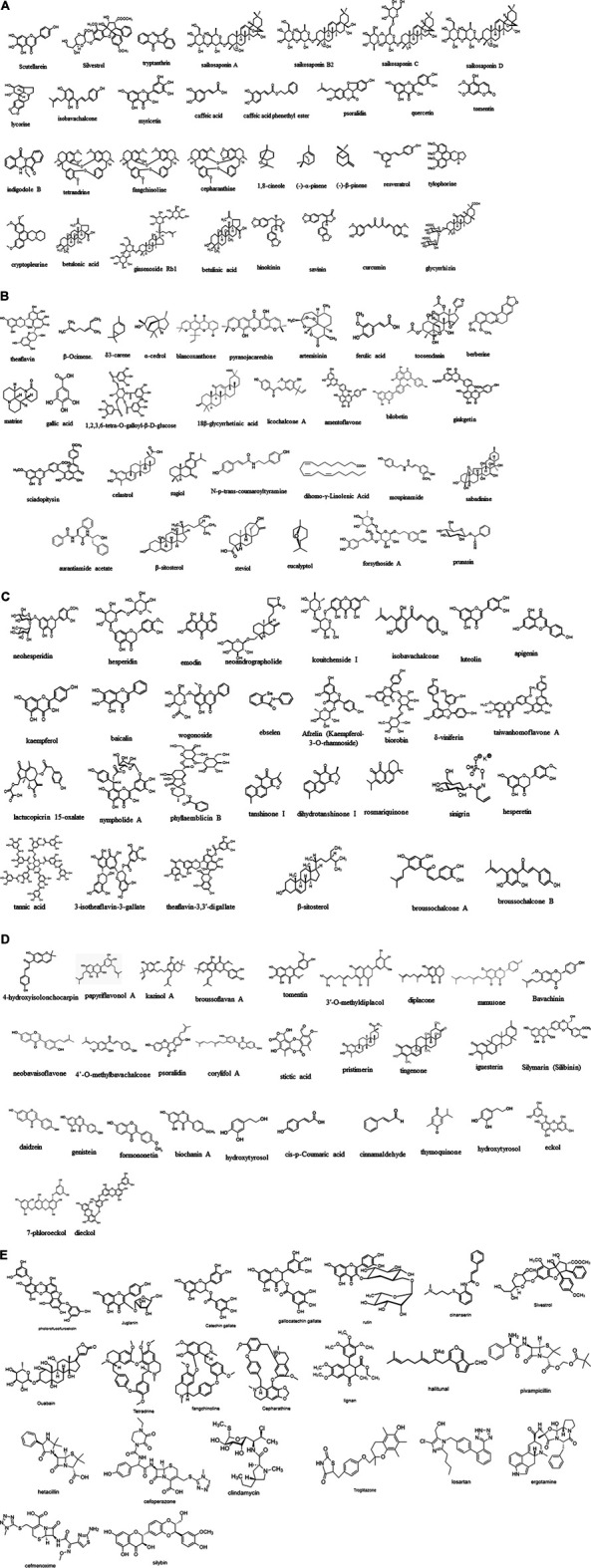
**(A–E)** Molecular structures experimentally effective for SARS-CoV therapeutic target. Scutellarein (CAS No: 529–53-3), silvestrol (CAS No: 697235-38-4), tryptanthrin (CAS No: 13220-57-0), saikosaponin A (CAS No: 20736-09-8), saikosaponin B2 (CAS No: 58316-41-9), saikosaponin C (CAS No: 20736-08-7), saikosaponin D (CAS No: 20874-52-6), lycorine (CAS No: 476-28-8), isobavachalcone (CAS No: 20784-50-3), myricetin (CAS No: 529-44-2), caffeic acid (CAS No: 331-39-5), caffeic acid phenethyl ester (CAS No: 104594-70-9), psoralidin (CAS No: 18642-23-4), quercetin (CAS No: 117-39-5), tomentin (CAS No: 28449-62-9), indigodole B, theaflavin (CAS No: 4,670–05-7), β-ocimene (CAS No: 3,338–55-4), δ3-carene (CAS No: 13,466–78-9), α-cedrol (CAS No: 77–53-2), blancoxanthone (PubChem CID: 11703574), pyranojacareubin (CAS No:78,343–62-1), artemisinin (CAS No: 63,968–64-9), ferulic acid (CAS No: 1,135–24-6), toosendanin (CAS No: 58,812–37-6), berberine (CAS No: 633–65-8), matrine (CAS No: 519–02-8), gallic acid (CAS No:149–91-7), 1.2,3,6-tetra-O-galloyl-β-d-glucose (CAS No: 79,886–50-3), 18β-glycyrrhetinic acid (CAS No: 471–53-4), licochalcone A (CAS No: 58,749–22-7), amentoflavone (CAS No:1,617–53-4), bilobetin (CAS No: 521–32-4), ginkgetin (CAS No: 481–46-9), sciadopitysin (CAS No: 521–34-6), celastrol (CAS No: 34,157–83-0), sugiol (CAS No: 511–05-7), N-*p*-trans-coumaroyltyramine (CAS No: 36,417–86-4), dihomo-γ-linolenic acid (CAS No: 1783–84-2), moupinamide (CAS No: 66,648–43-9), sabadinine (CAS No: 5,876–23-3), aurantiamide acetate (CAS No: 56,121–42-7), β-sitosterol (CAS No: 83–46-5), steviol (CAS No: 471–80-7), eucalyptol (CAS No: 470–82-6), forsythoside A (CAS No: 79,916–77-1), prunasin (CAS No: 99–18-3), tetrandrine (CAS No: 518–34-3), fangchinoline (CAS No: 436–77-1), cepharanthine (CAS No: 481–49-2), 1,8-cineole (CAS No: 470–82-6) (-)-α-pinene (CAS No: 7,785–70-8), (-)-β-pinene (CAS No: 18,172–67-3), ginsenoside Rb1 (CAS No: 41,753–43-9), resveratrol (*trans*-3,5,4′-trihydroxystilbene), homoharringtonine (CAS No: 501–36-0), tylophorine (CAS No: 482–20-2), cryptopleurine (CAS No: 482–22-4), betulonic acid (CAS No: 4,481–62-3), betulinic acid (CAS No: 472–15-1), hinokinin (CAS No: 26,543–89-5), savinin (CAS No: 493–95-8), curcumin (CAS No: 458–37-7), glycyrrhizin (CAS No: 1,405–86-3), neohesperidin (CAS No: 13,241–33-3), hesperidin (CAS No: 520–26-3), emodin (6-methyl-1,3,8-trihydroxyanthraquinone) (CAS No: 518–82-1), neoandrographolide (CAS No: 27,215–14-1), kouitchenside I (CAS No: 1444411-79-3), isobavachalcone (CAS No: 20,784–50-3), luteolin (CAS No: 491–70-3), 7-methylluteolin, apigenin (CAS No: 520–36-5), kaempferol (CAS No: 520–18-3), baicalin (CAS No: 491–67-8), wogonoside (CAS No: 51,059–44-0), ebselen (CAS No: 60,940–34-3), afzelin (CAS No: 482–39-3), biorobin (CAS No: 17,297–56-2), δ-viniferin (CAS No: 253,435–07-3), taiwanhomoflavone A (CAS No: 265,120–00-1), lactucopicrin 15-oxalate (CAS No: 303,130–75-8), nympholide A (CAS No: 604,004–58-2), phyllaemblicin B (CAS No: 307,504–07-0), tanshinone I (CAS No: 568–73-0), cryptotanshinone (CAS No: 35,825–57-1), dihydrotanshinone I (CAS No: 87,205–99-0), rosmariquinone (CAS No: 27,210–57-7), tannic acid (CAS No: 1,401–55-4), 3-isotheaflavin-3-gallate (CAS No: 30,462–34-1), theaflavin-3,3′-digallate (CAS No: 33,377–72-9), sinigrin (CAS No: 3,952–98-5), hesperetin (CAS No: 520–33-2), β-sitosterol (CAS No: 83–46-5), bavachalcone (CAS No: 28,448–85-30), broussochalcone A (CAS No: 99,217–68-2), broussochalcone B (CAS No: 28,448–85-3), 4-hydroxyisolonchocarpin (CAS No: 41,743–38-8), papyriflavonol A (CAS No: 363,134–28-5), 3′-(3-methylbut-2-enyl)-3′,4,7-trihydroxyflavane, kazinol A (CAS No: 99,624–28-9), kazinol B (CAS No: 99,624–27-8), kazinol F (CAS No: 104,494–35-1), kazinol J (CAS No: 104,778–05-4), broussoflavan A (CAS No: 99,217–69-3), tomentin A/B/C/D/E (CAS No: 36,034–36-3), 3′-O-methyldiplacol, 4′-O-methyldiplacol, 3′-O-methyldiplacone (CID No: 14,539,951), 4′-O-methyldiplacone (CID No: 24,854,122), mimulone (CAS No: 97,126–57-3), diplacone (CAS No: 73,676–38-7), bavachinin (CAS No: 19,879–30-2), neobavaisoflavone (CAS No: 41,060–15-5), isobavachalcone (CAS No: 20,784–50-3), 4′-O-methylbavachalcone (CAS No: 0,784–60-5), psoralidin (CAS No: 18,642–23-4), corylifol A (CAS No: 775,351–88-7), stictic acid (CAS No: 549–06-4), pristimerin (CAS No: 1,258–84-0), tingenone (CAS No: 50,802–21-6), iguesterin (CAS No: 53,527–47-2), silymarin (silibinin) (CAS No: 22,888–70-6), daidzein (CAS No: 486–66-8), genistein (CAS No: 446–72-0), formononetin (CAS No: 485–72-3), biochanin A (CAS No: 491–80-5), linolenic acid (CAS No: 463–40-1), palmitic acid (CAS No: 57–10-3), hydroxytyrosol (CAS No: 10,597–60-1), *cis*-*p*-coumaric acid (CAS No: 501–98-4), cinnamaldehyde (CAS No: 14,371–10-9), thymoquinone (CAS No: 490–91-5), hydroxytyrosol (CAS No: 10,597–60-1), eckol (CAS No: 88,798–74-7), 7-phloroeckol, dieckol (CAS No: 88,095–77-6), phlorofucofuroeckoln (CAS No: 128,129–56-6), juglanin (CAS No: 5,041–67-8), catechin gallate (CAS No: 1,257–08-5), (−)-gallocatechin gallate (CAS No: 5,127–64-0), rutin (CAS No: 153–18-4), cinanserin (CAS No: 1,166–34-3), sivestrol (CAS No: 697,235–38-4), ouabain (CAS No: 630–60-4), tetrandrine (CAS No: 518–34-3), fangchinoline (CAS No: 33,889–68-8), cepharanthine (CAS No: 481–49-2), diterpene (CAS No: 28,957–10-0), diterpene aldehyde, sesquiterpene (CAS No: 72,826–63-2), triterpene (CAS No: 125,343–14-8), lignan (CAS No: 6,549–68-4), halitunal (CAS No: 133,076–08-1), antibacterial agents such as pivampicillin (CAS No: 33,817–20-8), hetacillin (CAS No: 3,511–16-8), cefoperazone (CAS No: 62,893–19-0), clindamycin (CAS No: 18,323–44-9), antidiabetic drug troglitazone (CAS No: 97,322–87-7), antihypertensive drug losartan (CAS No: 114,798–26-4), analgesia drug ergotamine (CAS No: 113–15-5), antibacterial drug cefmenoxime (CAS No: 65,085–01-0), and hepatoprotective drug silybin (CAS No: 22,888–70-6).

Natural compounds including chemoenzymatic modified molecules can be used in ethnopharmacology to modulate SARS-CoV-2/nCoV-19 infections due to current limited therapeutic options. The efficacy of natural products depends on the CoV strains. Several natural products inhibit viral replication (Müller et al., 2020), implying antiviral properties. (Kalhori et al., 2021) For example, several compounds exhibit promising prospects for CoV treatment in human patients, as described above for lycorine, scutellarein, silvestrol, tryptanthrin, saikosaponin B2, and polyphenolic compounds such as caffeic acid, isobavachalcone, myricetin, psoralidin, and quercetin, as well as lectins such as griffithsin. For example, *Lycoris radiata* (L’Hér.) Herb lycorine shows cytopathogenic and antiviral activities against SARS-associated CoV (Yu et al., 2012). Currently known natural products that are pharmacologically effective for SARS-CoV-2 inhibition are shown in Figure 2, with the synthetic compounds previously utilized for other targets in humans.

Natural products that show viral inhibitory activity are promising candidates as anti-CoV agents. Natural products to combat the CoVs are reviewed in this article, focusing on the general properties of CoVs and suggesting applicable drugs and natural compounds effective against several CoV species. Viral proteins such as 3CLpro, PLpro, N, S, and ACE2 have been targeted for antiviral replication or anti-infection. Some limited antiviral agents as inhibitors specific for proteases and RNA synthases are known to block viral replication (Häkkinen et al., 1999). CoV bioactive natural products can also enhance and strengthen host immunity. Vitamins A and C lower susceptibility to infections and help in the prevention of viral infections through host immune function (Häkkinen et al., 1999).

### Inhibition of Ribonucleic Acid Helicase eIF4A and Protein Expression

SARS-CoV helicase, a virus replication enzyme, is involved in the unwinding of RNA. Helicases in protein sequences are commonly conserved during evolution in CoVs and other related nidoviruses. CoV helicase is an important therapeutic target because it hydrolyzes all deoxyribonucleotide and ribonucleotide triphosphates in SARS-CoV. Therefore, SARS-CoV-2 helicase has been targeted to screen for inhibitors. The 420-amino acid–long helicase is phylogenetically homologous to the helicases of other CoVs. Favipiravir or hydroxychloroquine, described later in further detail, recognizes SARS-CoV-2 helicase with weak affinities.

*Aglaia* sp. silvestrol inhibits the replication of MERS types with an EC50 value of 1.3 nM, acting as an inhibitor of RNA helicase eIF4A and protein expression via blocking replication/transcription complex formation (Miean and Mohamed, 2001). Silvestrol inhibits HcoV-229E protein synthesis with an EC50 of 3 nM. Silvestrol also inhibits HCoV-229E *ex vivo* in bronchial epithelial cells via RNA helicase eIF4A inhibition (Lau et al., 2008). The polyphenolic compounds myricetin and scutellarein inhibit the helicase activity of SARS-CoVs. Phenolic compounds including myricetin and scutellarein of *Isatis indigotica* Fort. and *Torreya nucifera* L. inhibit SARS-CoV helicases including nsP13 helicase (Cho et al., 2013). *Scutellaria baicalensis* Georgi (Scutellaria radix) myricetin and scutellarein inhibit the ATPase activity of the SARS-CoV helicase Nsp-13 (Yu et al., 2012). Myricetin is enriched in fruits such as cranberry *Vaccinium oxycoccos* L. (Mikulic-Petkovsek et al., 2012) and in vegetables such as *Calamus scipionum* Lam. and garlic (Qing et al., 2016). Scutellarein from *S. baicalensis* is a strong inhibitor of SARS-CoV helicase because it inhibits SARS-CoV helicase Nsp13 via ATPase activity inhibition but not via direct inhibition of helicase activity. The flavonoid quercetin is structurally similar to other polyphenolics such as myricetin and scutellarein and shows similar inhibitory activity of SARS-CoV helicase (Yang et al., 2010). In addition, naturally occurring tomentins of *Paulownia tomentosa* (Thunb.) Steud., belonging to Scrophulariaceae, reversibly and allosterically inhibit the PLpro activity of SARS-CoV (Lung et al., 2020).

### Inhibition of Ribonucleic Acid Genome Synthesis and Replication

RdRp, also known as nsp12, synthesizes a complementary RNA strand by using the original virus RNA genome as template. Inhibition of the SARS-CoV-2 RdRp enzyme is a potential therapeutic option for COVID-19 patients. Tryptanthrin inhibits viral RNA synthesis and PLpro-2 enzyme activity, important for the early, late, and post-entry step of HCoV replication. *Strobilanthes cusia* (Nees) Kuntze tryptanthrin, an indoloquinazoline moiety–carrying alkaloid, and the indigodole B (5aR-ethyltryptanthrin) alkaloid (Wen et al., 2007) have anti–HCoV-NL63 activity. *S. cusia* tryptanthrin and indigodole B also block RNA synthesis and PLpro-2 enzyme activity. Tryptanthrin is effective against SARS-CoV-2 and other HCoVs. The antiviral EC50 values were 1.52 µM for tryptanthrin and 2.60 µM for indigodole B. Tryptanthrin also has multiple pharmacological activities (Chen et al., 2008). In addition, tryptanthrin and indigodole B have direct antiviral activities to HCoV-NL63. *Houttuynia cordata* Thunb. aqueous extracts inhibit the enzyme activities of the viral 3CL protease and viral RdRp of SARS-CoV (Hoever et al., 2005). In computer modeling, *Camellia sinensis* (L.) Kuntze theaflavin (TF), 3,4,5-trihydroxy-1,8-bis [(2R,3R)-3,5,7-trihydroxy-2-chromanyl]-6-benzo (Kim, 2020) annulenone (C_29_H_24_O_12_), has been demonstrated to interact with the SARS-CoV-2 RdRp (Zhang et al., 2020). The TFs, known as antioxidant polyphenols, are formed from the precursor flavan-3-ols, which occur in green tea leaves, via condensation and enzymatic oxidation. Several derivatives, including TF-3′-gallate, TF-3-gallate, and TF-3–3′-di-gallate, are known. The TFs belong to the thearubigins, polymeric polyphenols, which show a red color, with a tropolone moiety.

Similarly, redwood *Sequoia sempervirens* (D.Don) Endl. natural phenol ferruginol compounds have various terpenoid substructures, such as betulonic acid [C_30_H_46_O_3_; 3-oxolup-20 (29)-en-28-oic acid; CAS No: 4481-62-3; CID 122844] and betulinic acid [C_30_H_48_O_3_; (3β)-3-hydroxy-lup-20 (29)-en-28-oic acid; CAS No: 472-15-1; CID 64971]; 8β-hydroxyabieta-9 (11),13-dien-12-one; 3β,12-diacetoxyabieta-6,8,11,13-tetraene; curcumin; hinokinin; and savinin. These compounds inhibit the replication of SARS-CoV (Loizzo et al., 2008). *Toona sinensis* (Juss.) M.Roem. extracts inhibit SARS-CoV replication (Jeong et al., 2014). Glycyrrhizin inhibits the replication of SARS-CoV after viral entrance into Vero cells, inhibiting virus attachment and entry (Shen et al., 2005). Several glycyrrhizin-derived compounds inhibit SARS-CoV replication more effectively (EC50 of 5–50 μM), but they are highly cytotoxic to Vero cells (Hoever et al., 2005). Lignin, betulinic acid, and desmethoxyreserpine inhibit viral replication as well as 3CLpro (Cheng et al., 2006). Especially desmethoxyreserpine blocks virus entry. *Laurus nobilis* L. and *Thuja orientalis* (L.) Franco essential oils are also inhibitors of viral replication. For example, *L. nobilis* β-ocimene (3,7-dimethyl-1,3,6-octatriene; CAS 502-99-8), 1,8-cineole (1,3,3-trimethyl-2-oxabicyclo-2.2.2-octane; CAS No: 470-82-6), *α*-pinene, and *β*-pinene, as well as *T. orientalis α*-pinene, δ3-carene, and *α*-cedrol inhibit viral replication (McDonagh et al., 2014).

β-Ocimene acts as a plant defense and antifungal agent and is derived from the plant genus *Ocimum*. Pinene (C_10_H_16_) is a bicyclic monoterpene. 1,8-Cineole oil, known as eucalyptol, is a cyclic ether and a monoterpenoid. Its synonyms are cajeputol; 1,8-epoxy-*p*-menthane; 1,8-oxido-*p*-menthane; and 1,3,3-trimethyl-2-oxabicyclo octane (Chan et al., 2020; Chan et al., 2020; Chan et al., 2020). *a*-Pinene is also a major constituent of the essential oil of *Rosmarinus officinalis* L (rosemary). Two enantiomers (1S,5S)- or (−)-α-pinene and (1R,5R)- or (+)-α-isomer are present. δ3-Carene or 3-carene is also a bicyclic monoterpene and has a pungent odor. *α*-Cedrol is a sesquiterpene alcohol and an essential oil component. It is an antioxidant with antiseptic, anti-inflammatory, anti-spasmodic, tonic, astringent, diuretic, sedative, insecticidal, and antifungal activities (Kim et al., 2008) and has been used in traditional medicine. *Calophyllum blancoi* Planch. & Triana pyranoxanthones such as blancoxanthone (C_23_H_22_O_5_; 5,10-dihydroxy-2,2-dimethyl-12-(2-methylbut-3-en-2-yl)-2H,6H-pyrano[3,2-b]xanthen-6-one) and pyranojacareubin inhibit HCoV-229E–infected host cell growth (Kim et al., 2010). The pyranoxanthones tested against HCoV-229E reverse *in vitro* virus-induced cytopathic effects. Blancoxanthone exhibits viral inhibition at 3 ug/ml in MRC-5 cells. *Bupleurum* sp., *Heteromorpha* sp., and *Scrophularia scorodonia* L. saikosaponin compounds interfere with the early replication step and entrance of HCoV-229E (Ulasli et al., 2014).

For animal CoVs such as feline CoVs, LMW molecules including artemisinin, baicalin, curcumin, quercetin, rutin, glycyrrhizic acid, hesperidin, and hesperitin inhibit replication of feline viruses (FCoVs) such as feline infectious peritonitis virus (FIPV)-1146 and FECV1683 via cytotoxicity of the virus-infected cells (O’Flaherty et al., 2019). For murine hepatitis virus (MHV) CoVs, ferulic acid, isoferulic acid, toosendanin, berberine, protoberberine alkaloids, matrine, oxymatrine, sophoranone, and sophocarpine isolated from *Cimicifuga racemosa* L., *Melia* sp., *Coptis* sp., *Phellodendron* sp., and *Sophora subprostrata* Chun & T. Chen. (Fabaceae) inhibit the replication of the murine hepatitis virus (MHV)-A59 strain (Song et al., 2014; Jin et al., 2019; Sun et al., 2019). Methanol extracts from the plants of *Sophorae* sp., *Acanthopanacis* sp., *Sanguisorbae* sp., and *Torilis* sp. possibly inhibit RdRp or other protease activity of MHV-A59. *Nigella sativa* L*.* and *Citrus sinensis* L. ethanol extract inhibits viral replication of MHV-A59 via an undetermined mechanism (Jin et al., 2019).

Artemisinin, isolated from *Artemisia annua* L. in 1972 by Dr. Tu Youyou, co-recipient of the 2015 Nobel Prize in Medicine, is an anti–*Plasmodium falciparum* malaria drug. It is a sesquiterpene lactone with an endoperoxide 1,2,4-trioxane ring, which is necessary to exert its activity. Artemisinin is a potential therapeutic candidate for certain RNA viruses (de Vries et al., 1997). Ferulic acid, as a hydroxycinnamic acid and a component of lignin, is a major metabolite of chlorogenic acid along with caffeic acid (CA) and isoferulic acid. Ferulic acid and its derivatives, including caffeoyltyramine, feruloyltyramine, and feruloyloctopamine, also inhibit SARS-CoV PLpro activity (Li, 2015). Recently, Adem et al. (2020) described that CA derivatives such as khainaosides, 6-O-caffeoyl-arbutin, and vitexfolin have been suggested to be inhibitory candidates with higher binding activities than that of nelfinavir against SARS CoV-2 S-protein as well as Nsp15 and Mpro enzymes by using molecular docking simulation via Web engines named Toxtree and www.swissadme.ch (Adem et al., 2021). Toosendanin (C₃₀H₃₈O₁₁), a triterpenoid isolated from the bark of *Melia azedarach* L., has analgesic, insecticidal, anti-botulinum, antimicrobial, and anti-inflammatory activities, and antiviral RNA polymerase complex activities (Simmons et al., 2013). Matrine, an alkaloid of *Sophora flavescens* Aiton, inhibits IL-1β expression and MyD88/NF-κB and NLRP3 inflammasome in the inflammatory response in porcine respiratory syndrome virus–infected pigs (Hulswit et al., 2019). Using a structure- and activity-based computational approach, natural products have been analyzed for the Nsp-9 (PDB ID-6W4B) enzyme inhibition of SARS-CoV-2 RNA replication and S-protein binding (Chandel et al., 2020). Baicalin exhibits binding affinity to both S-protein and Nsp9 enzyme.

### Inhibition of N-Protein and S-Glycoprotein Synthesis and Replication

#### Modulation of S-Glycoprotein

S-glycoprotein recognizes the host cell receptor to enter the cells through endosomal fusion, after which the S-glycoprotein is cleaved, endosomal membranes released, and RNA liberated into the cytosol (Langereis et al., 2012). S-glycoprotein interacts with its receptors via its RBD and plays a role in host tropism and pathogenicity, and in proposing some therapeutic clues (Xiong et al., 2013). Therefore, modulation of the S-glycoprotein is a potential target to control SARS-CoV-2 propagation. Tetrandrine, fangchinoline, and cepharanthine as bis-benzylisoquinoline alkaloids from *Stephania tetrandra* var. *glabra* Maxim. protect cells from virus-induced cell death. In addition, they inhibit viral replication, as well as CoV S-glycoprotein and N-protein synthesis. Also, they induce the virus-induced host response by the p38MAPK pathway. Terpenoid compounds such as *α*- and *β*-pinene as well as cineole interact with the infectious bronchitis virus (IBV) N-protein to inhibit the N-protein–RNA interaction and block IBV replication. The terpenoids bind to the N-terminal active site of the N-protein. The active site is composed of five amino acid residues (Yang et al., 2011; Müller et al., 2020). These conserved amino acids in the active sites are commonly located in various IBVs. CA from *Sambucus javanica* subsp. *chinensis* Fukuoka (elderberry) extract inhibits the HCoV strain HCoV-NL63 (Weng et al., 2019). CA inhibits HCoV S-glycoprotein attachment to host cells. Chlorogenic acid and gallic acid (3,4,5-trihydroxybenzoic acid) also exhibit the same activities as CA. Gallic acid is a trihydroxybenzoic acid and forms dimeric ellagic acid. Tannins are hydrolyzed to glucose and gallic acid (gallotannin), or glucose and ellagic acid (ellagitannin). CA also inhibits the hepatitis B virus (Wang et al., 2009). *Sambucus nigra* L. extract (black elderberry) has been used for treating cold and flu symptoms. The adsorption, bioavailability, metabolism, and delivery mechanism of the extracts are documented for therapeutic plasma concentrations (Wittemer et al., 2005).

Four saikosaponins inhibit human CoV-229E infectivity. These saikosaponin pentacyclic triterpenoid glycoside derivatives purified from *Bupleurum* spp., *Heteromorpha* spp., and *S. scorodonia* L. also have anti-HIV and anti-HCoV-22E9 activities *in vitro* (Ushio and Abe, 1992; Chiang et al., 2003; Ulasli et al., 2014). Saikosaponins show anti-CoV activity, and saikosaponin B2 also shows the highest potency with an EC50 of 1.7 µM and inhibits the early stage of CoV viral attachment to host receptors via S-glycoprotein and penetration into the cells. The *Streptomyces parvulus* actinomycin D antibiotic also inhibits CoV attachment and penetration stages (O’Flaherty et al., 2019). *Panax ginseng* (T.Nees) C.A. Mey. ginsenoside Rb1 (gynosaponin C) as steroidal glycosides and triterpene saponins exhibit antiviral activity (Wu et al., 2004). *Stephania tetrandra* var. *glabra* bis-benzylisoquinoline alkaloid compounds such as tetrandrine, fangchinoline, and cepharanthine exhibit antiviral activity on HCoV-OC43 (Kim et al., 2019). They inhibit virus-induced cell death via blocking of virus replication and S-glycoprotein and N-protein synthesis with the host response. Resveratrol (CAS number: 501–36-0), a stilbenoid, inhibits MERS-CoV replication and infection in a cell-based system by inhibition of MERS-CoV N-protein expression and MERS-CoV–induced host cell death (Lin et al., 2017). Resveratrol inhibits SARS-CoV-2 infection.

The NIH clinical collection of 727 tested antiviral compounds showed that the alkaloid omacetaxine (homoharringtonine) shows a nonomolar IC50 level (Cao et al., 2015). Two alkaloids of *Tylophora indica* (Burm. f.) Merr., tylophorine and 7-methoxycryptopleurine, inhibit transmissible gastroenteritis CoV replication (Yang et al., 2010). The *T. indica* alkaloids tylophorine and 7-methoxycryptopleurine block replication in CoV-infected cells of swine testicular tissues (Cho et al., 2006). 7-Methoxycryptopleurine (IC50 of 20 nM) is rather more efficient than tylophorine (IC50 of 58 nM). Tylophorine also blocks virus RNA replication and NF-κB activation mediated by cellular JAK phosphorylation in CoV (Yang et al., 2017). Tylophorine and 7-methoxycryptopleurine inhibit N- and S-glycoprotein activity. Dihydrotanshinone recognizes the S-glycoprotein of SARS-CoV-2 to block its entry (Zhang et al., 2020). *Rhus chinensis* Mill. luteolin and tetra-O-galloyl-β-d-glucose (TGG) specifically recognize the S2 subunit and prevent viral entry of SARS-CoV (Yi et al., 2004). Luteolin also binds to the S2 protein to exert its antiviral capacity by interfering with virus–cell attachment and consequent fusion. TGG and luteolin exhibit anti–SARS-CoV activities. Therefore, LMW natural products, which bind to the SARS-CoV S-glycoprotein, can block virus infection in its host cells.

For animal CoVs such as avian IBV, *Alstonia scholaris* (L.) R. Br. alstotide-1 and -3 interfere with membrane proteins and S-glycoproteins but not the nucleocapsid proteins of avian IBV (Nguyen et al., 2015). These peptide-derived drugs are potentially applicable for therapeutic characteristics, although they are poor in oral bioavailability. However, alstotides are suggested to be permeable to cells, stable, and nontoxic with anti-IBV activities. Alstotide-1 interacts with the IBV M-protein during the assembly and budding of virus particles. M-protein is a glycosylated and membrane-spanning protein. The alstotide-1 and M-protein interaction implicates that alstotide-1 inhibits the assembly and budding of virus particles. *Punica granatum* L. polyphenols also interact with the surface S-glycoprotein of murine CoV, MHV-A59 (Sundararajan et al., 2010).

#### Inhibition of Interaction of S-Glycoprotein With ACE2

The β-CoV SARS-CoV recognizes ACE2 in respiratory epithelial or type I and II alveolar epithelial cells of the lung in membrane-bound and soluble forms (Alifano et al., 2020). ACE2 is a type I membrane-anchored carboxypeptidase with an N-terminal signal peptide. The host SARS-CoV-2 receptor ACE2 in the renin–angiotensin system (RAS) plays a role in lung infection through removal of the barrier. The SARS-CoV-2 S-glycoprotein recognizes ACE2. ACE2 is necessary for a virus receptor. The receptor-binding motif (RBM) recognizes human ACE2 (Li, 2015; Gheblawi et al., 2020). The α-CoV HCoV-NL63 and the lineage B *β*-CoV SARS-CoV S-glycoproteins are well known to bind to ACE2, but *β*-CoV MERS virus is not specific for the ACE2 recognition, while the *α*-CoV HCoV-NL63 is specific for the ACE2 recognition. Thus, the S viral protein drives the first attachment step on respiratory cell surfaces. This is a therapeutic target. Host ACE2 is the known host site for the S-glycoprotein RBD. The RBD sequence of the SARS-CoV-2 S-glycoprotein is homologous to the RBD of the SARS-CoV S-glycoprotein. ACE2 is also a SARS-CoV-2 drug target. To date, the ACE2 protein can be recognized by the antidiabetic troglitazone, antihypertensive losartan, anti-analgesic ergotamine, antibacterial cefmenoxime, and hepatic-protective silybin. *Phyllanthus emblica* L. phyllaemblicin G7, the genus *Swertia*, *Citrus aurantium* L. xanthones, neohesperidin, and hesperidin bind to the ACE2 protein, but not to the ACE2–S-protein RBD interface. A flavonoid hesperidin isolated from citrus peel interacts with the SARS-CoV-2 receptors (Meneguzzo et al., 2020). In molecular docking analysis, flavonoids and anthraquinones exhibit binding capacities to ACE2. Their binding sites of ACE2 protein are different from that of the viral S-protein. For example, the flavone chrysin (CID: 5281607) isolated from the medicinal plant *Oroxylum indicum* binds to the ACE2 protein *in silico* (Basu et al., 2020).

The S-glycoprotein cleavage TMPRSS2 enzyme potentiates SARS- and MERS-CoVs infections. Several antibacterial agents such as pivampicillin, hetacillin, cefoperazone, and clindamycin, and antiviral kouitchenside I potentially inhibit the TMPRSS2 enzyme (Wu et al., 2020). The anthraquinone emodin (CAS No: 518-82-1) blocks the SARS-CoV S-glycoprotein and ACE2 interaction (Ho et al., 2007). Glycyrrhizin-modified compounds such as 18β-glycyrrhetinic acid are known to be anti–SARS-CoV agents due to their cytopathogenic effects (Haiying et al., 2003; Hoever et al., 2005). 18β-Glycyrrhetinic acid is a glycyrrhizin metabolite that is converted by intestinal microbes in humans and is an inhibitor of the complement cascade. 18β-Glycyrrhetinic acid inhibits DNA polymerases and suppresses TNF-α expression. Glycoside chain modification of glycyrrhizin with 2-acetamido-β-d-glucopyranosylamine increased its antiviral activity by 10-fold, through increased interaction with the S-glycoprotein. Glycyrrhizin and its derivatives, such as 18β-glycyrrhetinic acid and licochalcone A, are constituents of *Glycyrrhiza uralensis* Fisch. (licorice), *G. glabra* L., or *G. inflata* Bat. (Fu et al., 2016).

In addition, 18β-glycyrrhetinic acid and licochalcone A bind to the ssRNA virus nucleoprotein (NP), a target candidate for therapeutic development, because these natural ligands influence the RNA-binding property of NP. The two agents specifically recognize the RNA-binding groove of NP (PDB code 4Z9P) and disrupt the NP–viral ssRNA interaction through a conformational shift of NP oligomers to impair ssRNA assembly. Glycyrrhizin and glycyrrhetinic acid also block SARS-CoV replication (Cinatl et al., 2003). In addition, glycyrrhizin inhibits H5N1 influenza A virus replication (Michaelis et al., 2011). Glycyrrhizin and glycyrrhetinic acid are also anti-inflammatory, antiviral, and anti-allergic agents. Licochalcone A is a natural phenolic chalconoid found in the *Glycyrrhiza* species and exhibits antimalarial and antiviral activities. It inhibits influenza neuraminidases (NAs) of influenza subtypes such as H1N1, H9N2, and oseltamivir-resistant novel H1N1strains (Chen et al., 1994; Dao et al., 2011).

### Inhibition of NLRP3 Inflammasome Signaling

SARS-CoV protein domains modulate NLRP3 inflammasome–triggered pulmonary inflammation via chemokines. Therefore, the NLRP3 inflammasome is a potential candidate for therapeutic agents against CoV-mediated inflammatory diseases. Natural products such as flavonoids interfere with signaling mediated by the NLRP3 inflammasome. Respiratory inflammatory SARS-CoVs induce the NLRP3 inflammasome in macrophages and Th1 cells. Several flavonoids inhibit NLRP3 inflammasome–related inflammatory response to SARS-CoVs. Such flavonoids include isobavachalcone, saikosaponin B2, silvestrol, tryptanthrin, CA, quercetin, myricetin, psoralidin, scutellarein, luteolin, apigenin, kaempferol, baicalin, and wogonoside (Sun et al., 2015; Fu et al., 2016; Choe and Kim, 2017; Lim et al., 2018; Zhang et al., 2018; Chen et al., 2019; Chen et al., 2019; Yamagata et al., 2019).

### Inhibition of SARS-CoV Mpro, PLpro, 3CLpro, and Related Proteases

The CoV genomes encode a polypeptide which contains a protease region. Two cysteine proteases, PLpro and 3CLpro, are directly associated with RNA virus replication. PLpro and 3CLpro cleave the viral polyprotein and produce nonstructural proteins for viral replication at the commonly conserved 11 substrate-recognition sites. Structure-based information on PLpro from SARS-CoV or other CoVs is limited. 3CLpro is also called the CoV main protease (MPro) (MW 34 kDa). Thus, Mpro is used as a target for anti-CoV drugs. Mpro controls overall RNA replication and transcription. Therefore, it is a target protease, and computational *in silico* simulation enables the discovery of SARS-CoV-2 Mpro-specific inhibitors (Jin et al., 2020). 3CLpro has 100% identity with other SARS-CoV genomic RNA sequences. The 3CLpro of bat and human SARS-CoV-2 exhibits 99.02% amino acid sequence homology. The SARS-CoV-2 3CLpro protein is homologous with the known SARS-CoV, HCoV, MERS-CoV, and BCoV.

Among the virus targets, CoV Mpro and ACE2 are the main targets to screen. An Mpro inhibitor N3 was designed by AI-driven drug simulation and docking. Michael acceptor inhibitor or N3 inhibits the SARS- and MERS-CoV Mpros through an inhibitory mechanism of irreversible covalent bond formation with Mpro (Jin et al., 2020). Additionally, another SARS-CoV-2 Mpro inhibitor, ebselen, was designed, with N3 and ebselen exhibiting antiviral activity against SARS-CoV-2. Also, multiple natural compounds including afzelin, biorobin, hesperidin, δ-viniferin, myricitrin, taiwanhomoflavone A, lactucopicrin 15-oxalate, nympholide A, and phyllaemblicin B recognize SARS-CoV-2 Mpro with additional binding activities to hACE-2 and RdRp (Rasool et al., 2020). In the molecular interaction of natural products with the Mpro docking pocket, *Psorothamnus arborescens* var. *simplifolius* (Parish) Barneby 5,7,3′,4′-tetrahydroxy-2’-(3,3-dimethylallyl) isoflavone (PubChem 11610052) showed a high binding affinity via rigid hydrogen bonds to the catalytic dyad amino acid residues, and binding to the RBD at the amino acid residues. Similarly, *Myrica cerifera* L. myricitrin (PubChem 5281673) and methyl rosmarinate showed RBD to and receptor binding affinities to SARS-CoV-2 Mpro in a stability assay of ligand–protein complex (Joshi et al., 2020). Withanolide derivatives such as withaferin isolated from *Ashwagandha* species and the CA derivative CA phenethyl ester (CAPE) exhibit binding capacities to Mpro enzyme. CAPE and withaferin recognize the SARS-CoV-2 Mpro SBD with equivalent potentials to the known N3 protease inhibitor, as analyzed using dynamic simulation (Kumar et al., 2020). As potential COVID-19 Mpro inhibitors, flavonoid derivatives including curcumin derivatives, apigenin derivatives, oleuropein, catechin derivatives, and kaempferol have been suggested as candidates in the docking analysis (Khaerunnisa et al., 2020). In a recent report using the docking analysis (Erlina et al., 2020), ermanin compound known as kaempferol-di-O-methyl ether, myricetin glycosides, peonidin arabinosides, quercetin rhamnosides, rhamnetin mannosyllsides, and hesperidin have also been suggested to exhibit SARS-CoV-2 protease inhibitory activities. A cyclic ether and monoterpenoid component, jensenone, which is found in eucalyptus plant oil, potentially inhibits Mpro enzyme activity (Sharma and Kaur, 2020). Similarly, phytochemicals such as *Allium cepa* L. oleanolic acids, *Cocos nucifera* L. α-tocotrienols, *Psidium guajava* L*.* asiatic acids, and *Eucalyptus globulus* culinosides exhibit anti–SARS-CoV-2 activity in molecular dynamic docking analysis. Oleanolic acid specifically binds to the Mpro enzyme (Fitriani et al., 2020).

The SARS-CoV PLpro cleaves junctions spanning Nsp1–Nsp4. PLpro also deubiquitinates proteins and helps the virus to evade the innate immune response (Ratia et al., 2008). Therefore, PLpro is a target for drug development against disease-associated deubiquitinating enzymes (Ghosh et al., 2010). Cinnamic amides isolated from *Tribulus terrestris* L. fruits inhibit PLpro activity (Song et al., 2014). Some plants including *Cassia tora* L., *Cibotium barometz* (L.) J. Sm., *Dioscorea polystachya* Turcz., *Gentiana scabra* Bunge, and *Taxillus chinensis* (DC.) Danser showed SARS-CoV 3CLpro enzyme activity (Wen et al., 2011). Natural compounds such as lignins, tannins, and coumarins have also been found to exhibit CoV inhibitory activities (Islam et al., 2020). The diterpenoid 8β-hydroxyabieta-9 (11),13-dien-12-one and a lignin compound savinin inhibited SARS-CoV 3CLpro activity. *Salvia miltiorrhiza* Bunge tanshinone I, IIA, IIB; dihydrotanshinone; methyl tanshinonate; cryptotanshinone; and rosmariquinone inhibited 3CLpro and PLpro with anti-infection and anti-replication activities, where tanshinone I and dihydrotanshinone I are strong 3CLpro and PLpro inhibitors (Park et al., 2012). The above *S. miltiorrhiza* Bunge tanshinone derivatives are noncompetitive inhibitors of protease enzyme isomerization. Especially, rosmariquinone reversibly inhibits the slow binding during cysteine protease isomerization (Park et al., 2012).

Black tea phenolic components such as tannic acid, 3-isotheaflavin-3-gallate, and theaflavin-3,3′-digallate also exhibited 3CLpro inhibition (Chen et al., 2005). *Isatis indigotica* Fortune ex Lindl. phenolic compounds including sinigrin, indigo, emodin, hesperetin, and β-sitosterol inhibited 3CLpro activity (Lin et al., 2005). *Torreya nucifera* (L.) Siebold & Zucc. flavones, biflavones, amentoflavone, apigenin, luteolin, bilobetin, ginkgetin, sciadopitysin, and quercetin also inhibit SARS-CoV 3CLpro (Ryu et al., 2010). Amentoflavone, bilobetin, ginkgetin, and sciadopitysin biflavonoids are constituents of *Ginkgo biloba* L. Other plants such as *Chamaecyparis obtusa* (Siebold & Zucc.) Endl. and *Hypericum perforatum* L. are also known to contain these compounds. They inhibit the cathepsin B inhibitor and influenza virus NA. Amentoflavone, bilobetin, ginkgetin, and sciadopitysin are noncompetitive inhibitors of CoV 3CLpro (Ryu et al., 2010). Myricetin and scutellarein inhibit SARS-CoV 3CLpro activity (Cho et al., 2013). *Broussonetia papyrifera* (L.) L'Hér. ex Vent. broussochalcone A/B, kazinol A/B/F/J, broussoflavan A, 4-hydroxyisolonchocarpin, papyriflavonol A, and 3′-(3-methylbut-2-enyl)-3′,4,7-trihydroxyflavane inhibit the 3CLpro and PLpro enzymes, where papyriflavonol A is the best inhibitor of the PLpro enzyme (Ryu et al., 2010). *B. papyrifera* 3′-(3-methylbut-2-enyl)3′,4,7-trihydroxyflavane noncompetitively inhibits PLpro activity (Park et al., 2017). However, these compounds do not inhibit PLpro of MERs-CoV, indicating strain dependence. Other polyphenolic compounds such as kazinol F and broussochalcone A of the same *B. papyrifera* inhibit MERS-CoV PLpro.

*Paulownia tomentosa* tomentins 3′-O-/4′-O-methyldiplacol, 3′-O-/4′-O-methyldiplacone, mimulone, diplacone, and 6-geranyl-4′,5,7-trihydroxy-3′,5′-dimethoxyflavone inhibit PLpro of SARS-CoV (Lung et al., 2020). *Psoralea corylifolia* L. polyphenolics including bavachinin, neobavaisoflavone, isobavachalcone, 4′-O-methylbavachalcone, psoralidin, and corylifol A inhibit PLpro activity. Among these, isobavachalcone and psoralidin exhibit the highest antiviral activity with reversible inhibitory activity against PLpro via a type I mechanism (Kim et al., 2014). Quercetin inhibits PLpro and 3CLpro proteases (Ryu et al., 2010). For porcine epidemic diarrhea virus (PEDV), quercetin-7-rhamnoside, a disaccharide glucoside, inhibits viral activity rather than quercetin alone. In a computer-based simulation for a protease 3CLpro inhibitor of feline CoVs, naturally occurring compounds such as 7-methylluteolin, stictic acid, and quercetin-7-rhamnoside showed inhibition. However, only stictic acid prevented virus-induced death and virus attachment to the host cells, while 7-benzyl luteolin and steviol showed no inhibitory effects (Theerawatanasirikul et al., 2020). Similarly, quercetin-7-rhamnoside exhibits higher antiviral activity against animal CoVs than quercetin (Choi et al., 2009). *Psoralea corylifolia* bavachinin, neobavaisoflavone, isobavachalcone, 4′-O-methylbavachalcone, psoralidin, and corylifol inhibited PLpro of SARS-CoV (Lin et al., 2005). Interestingly, psoralidin strongly inhibited the protease activity of SARS-CoV. Using an *in vitro* cell-based assay of Vero E6 cells, terpenoids and lignoids were shown to block 50% cell growth of Vero E6 cells infected with SARS-CoV. Betulinic acid and savinin competitively inhibit SARS-CoV 3CLpro with a Ki of 8.2 and 9.1 μM, respectively (Loizzo et al., 2008). Quinone-methide triterpenes such as celastrol (tripterine), pristimerin, tingenone, and iguesterin of *Triterygium regelii* Sprague & Takeda inhibit the 3CLpro activity (IC50 = 5.5, 9.9, and 2.6 μM) as competitive inhibitors (Ryu et al., 2010). Celastrol as a pentacyclic triterpenoid in quinone methides also inhibits the RNA of hepatitis C and dengue viruses (Tseng et al., 2017; Yu et al., 2017). *Torreya nucifera* ethanolic extract contains SARS-CoV 3CLpro inhibitors. Biflavone amentoflavone was identified as a potent 3CLpro inhibitor via molecular docking (Ryu et al., 2010). Geranylated flavonoids are the strongest inhibitors of PLpro activity (Lung et al., 2020).

Sugiol, coumaroyltyramine, N-cis-feruloyltyramine, kaempferol, quercetin, cryptotanshinone, and tanshinone IIA inhibit PLpro and 3CLpro. Dihomo-γ-linolenic acid and moupinamide (feruloyltyramine) inhibit 3CLpro and PLpro, respectively (Cheng et al., 2006). Through computer docking modeling of SARS-CoV 3CLpro, *Veratrum sabadilla* Retz. sabadinine inhibits CoV protease (Toney et al., 2004). *Artemisia annua* aurantiamide acetate inhibits the active pocket of the CoV cathepsin-L protease (Wang et al., 2007). *Isatis indigotica* sinigrin, indigo, β-sitosterol, aloe-emodin, and hesperetin (Islam et al., 2020) as well as *Rheum palmatum* L. anthraquinones inhibit 3CLpro (Luo et al., 2009). *Houttuynia cordata* Thunb. water extract inhibits 3CLpro protease (Yang et al., 2010). For animal CoVs, stictic acid, 7-methylluteolin, quercetin-7-rhamnoside, 7-benzyl luteolin, and steviol, which exist in plants such as lichen, inhibit the 3CLpro protease activity of FIPV1146 (FCoV) (Theerawatanasirikul et al., 2020). *Uncaria tomentosa* (Willd. ex Schult.) DC, known as cat’s claw, exhibits 3CLPro inhibitory activity as found by molecular docking analysis (Yepes-Pérez et al., 2020). Isolated phytochemicals such as cadambine, speciophylline, and proanthocyanidin of *Uncaria tomentosa* effectively interacted with 3CLpro.

As described above, CoV proteases are considered antiviral targets for the reduction of virus replication and host pathogenicity. However, nM affinity–leveled compounds are not developed for the targets. Apart from the conventional discovery from natural products, computer-aided methods to design drugs have been applied by using chemical databases for the inhibitor screening of SARS-CoV 3CLpro activity. Moreover, the known crystal structure of HCoV-229E 3CLpro facilitates design of inhibitors (Anand et al., 2003). Currently, SARS-CoV 3CLpro (PDB: 1Q2W and 1UK4) and SARS-CoV 3CLpro are elucidated for their 3D structures (Yang et al., 2003).

### ADAM17 and TMPRSS2 Serine Protease Inhibitors

**FIGURE 3:**
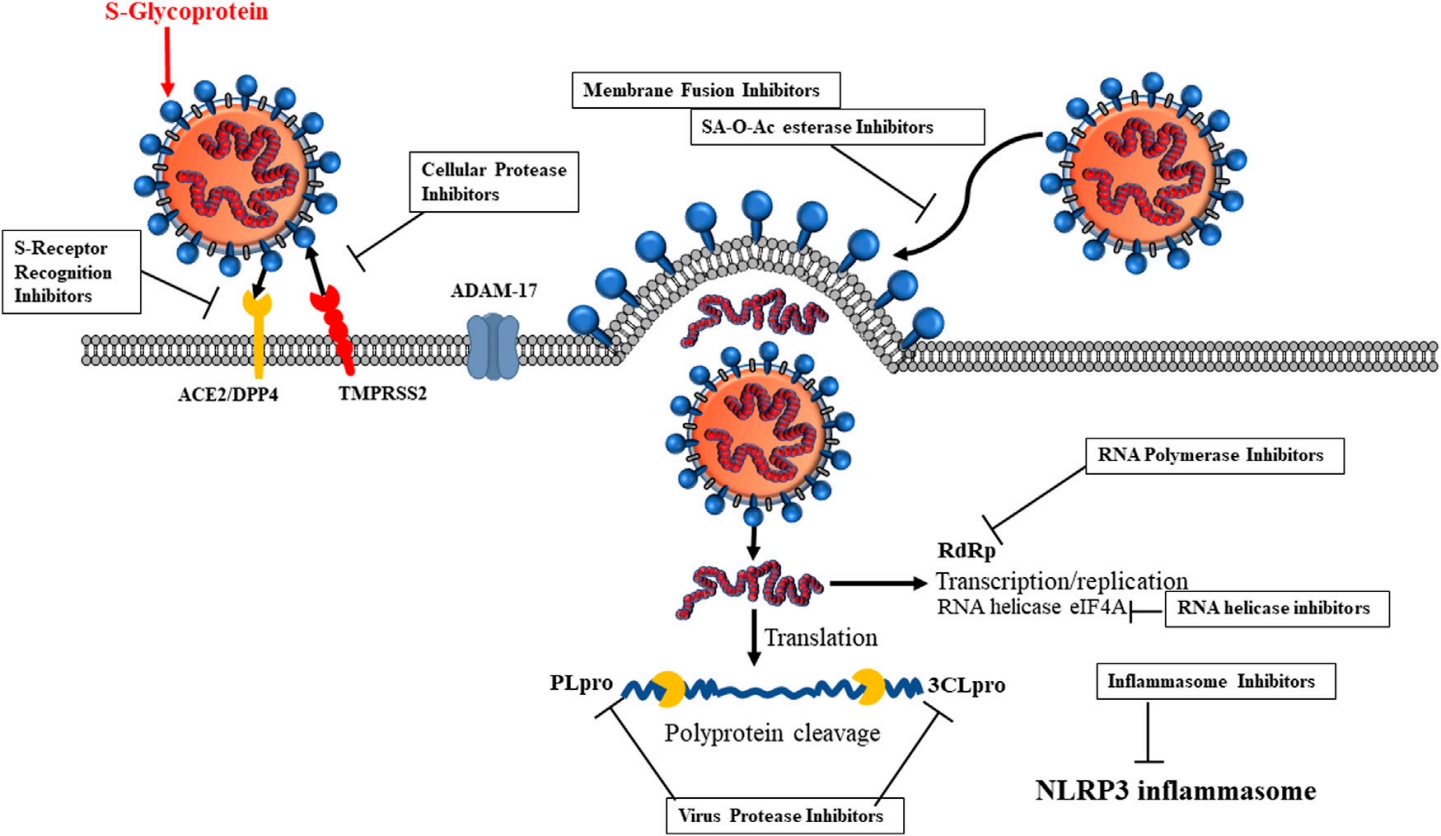
Inhibitor candidates for SARS-CoV-2 life cycle. 1) S-glycoprotein receptor recognition, 2) cellular protease, 3) virus proteases, 4) RNA polymerase, 5) inflammasome, 6) RNA helicase, 7) SA-O-Ac-esterase.

SARS-CoV-2 also utilizes TMPRSS2 for infection. A disintegrin and metallopeptidase domain (ADAM) family comprises Zn-metalloproteinases, and ADAM17 is a specific TNF-α–converting enzyme (TACE), thus also named TNF-α sheddase. ADAMs include ADAM-9, -10, and -12. ADAM17 sheds the ACE2 enzyme and catalyzes the formation of the soluble ACE2 N-terminal carboxypeptidase domain from ACE2 (Tipnis et al., 2000) and also converts pro-TNF-α to soluble TNF-α. Thus, ADAM17 is an anti-inflammatory target. Interaction of SARS S-glycoprotein and ACE2 cleaves ACE2 via ADAM17/TACE, facilitating its shedding and virus entry (Chen et al., 2008). TMPRSS2, human airway trypsin-like protease (HAT), TM protease, serine 13, serine protease DESC1, furin, factor Xa, and endosomal cathepsin L/B can cleave the SARS-CoV S-protein, facilitating SARS-CoV infection (Heurich et al., 2014). However, only TMPRSS2 allows SARS-CoV infection (Haga et al., 2010; Park et al., 2016; Zuniga et al., 2020). ACE2 interaction and TMPRSS2 activation potentiate the viral attachment to host cells. Thus, TMPRSS2 is a target for therapeutic agents (Figure 3).

SARS-CoV S-cellular TNF-α–converting enzyme activation facilitates virus entry, and thus this enzyme is an antiviral target. The inhibitor TAPI-2 inhibits virus entry of SARS-CoV into host cells. TAPI-2 inhibits SARS S-glycoprotein–mediated ACE2 shedding and TNF-α synthesis in the lung (Haga et al., 2010). ADAM17 inhibitors are widely beneficial for various diseases related to tumor immunosurveillance, cancer, and inflammatory diseases. As described previously, ADAM17 inhibitors reduce TNF-α–induced proinflammatory diseases and are attractive target candidates for the inflammatory diseases involved in SARS-CoVs. For example, a dual and selective small molecular inhibitor of ADAM17 and ADAM10, named INCB7839, is currently under combined usage with rituximab for B-cell non-Hodgkin lymphoma therapy (Witters et al., 2008). Although ADAM17 inhibitors such as matrix metalloproteinase (MMP) inhibitors marimastat and prinomastat inhibit ADAM17 activity (Packer and Cadenas, 2011), they are not clinically applicable due to ADAM17 sequence homology with the MMP enzymes and physiological problems. In this context, naturally occurring molecules have been used to attempt to develop selective ADAM17 inhibitors by using *in silico* approaches toward ligands and targets. Through the binding of ADAM17 to ligands, silymarin has been purified as an ADAM17-specific inhibitor that binds to the active amino acid residues in the ADAM17 protein. The inhibiting capacity has been compared with a previously known inhibitor, IK682. Silymarin is found in *Silybum marianum* (L.) Gaertn., known as milk thistle; and *Cynara cardunculus* L., known as wild artichokes; *Curcuma longa* L. turmeric rhizome; and *Coriandrum sativum* L. coriander seeds (Borah et al., 2016).

Cryptotanshinone, a natural compound isolated from *S. miltiorrhiza*, modulates androgen receptor (AR) transcriptional regulation and downregulates TMPRSS2 gene expression as an AR target gene in androgen-responsive tumor cells. Interestingly, cryptotanshinone selectively inhibits the AR gene and thus has potential as anti-AR or SARS-CoV therapy (Xu et al., 2012).

### Inhibition of GRP78 (HSPA5) Interaction *in Silico*

MERS-CoV spikes also recognize a 78-kDa glucose-regulated protein (GRP78) known as Byun1, heat shock 70-kDa protein 5 (HSPA5), and binding immunoglobulin protein (BiP). HSP5A is an ER-resident unfolded protein response (UPR) protein and acts as an alternative entry site via S-protein interaction for human viruses including papillomavirus, Ebola virus, Zika virus, and HCoVs, as well as the fungus *Rhizopus oryzae* (Pujhari et al., 2019; Elfiky, 2020; Elfiky, 2020; Ibrahim et al., 2020). Viral infection increases HSPA5 translocation to the plasma membrane (PM) and forms a membrane protein complex. In addition, GRP78 regulates MERS-CoV entry in the presence of DPP4. Lineage D β-CoV and bat CoV HKU also recognize the GRP78 (Ho et al., 2007; Yang et al., 2010), as simulated by molecular modeling and docking (Rao et al., 2002). Other ER molecules such as activating transcription factor 6 (ATF6), inositol-requiring enzyme 1 (IRE1), and protein kinase RNA (PKR)–like ER kinase (PERK) (Ibrahim et al., 2019) are involved. GRP78 releases IRE1, ATF6, and PERK activation, contributing to translation and refolding. GRP78 translocates to the membrane and recognizes the virus by its substrate-binding domain *β* (SBDβ), which is bound by the RBD. The binding region is molecularly targeted for COVID-19–specific drugs. Therefore, natural products can inhibit the HSPA5 binding to the S-glycoprotein. Small natural products prevent the S-glycoprotein–HSPA5 SBDβ interaction *in silico*. The effects of natural products that cause HSPA5 SDBβ dysfunction prevent SARS-CoV-2 S recognition because the HSPA5 SBDβ is the binding site for the SARS-CoV-2 S-glycoprotein. During viral infection, the HSPA5 (GRP78) translocated to the cell PM recognizes the SARS-CoV-2 S-protein. In *in silico* AI computer-aided simulation, several natural products recognize HSPA5 SBDβ. HSPA5 SBDβ-binding natural products can block virus attachment to the host cells if they are stressed. Thus, anti–COVID-19 agents specific for HSPA5 SBDβ recognition can be beneficial for elderly humans with cell stress. Therefore, approaches using AI computer-based molecular docking simulation yielded some natural products that bind to HSPA5 SBDβ (Elfiky, 2020). Four *Cicer arietinum* L. phytoestrogens, daidzein, genistein, formononetin, and biochanin A, recognize HSPA5 SBDβ. In addition, other natural compounds such as chlorogenic acid, linolenic acid, palmitic acid, CA, CA-phenethyl ester (CAPE), hydroxytyrosol, *cis*-*p*-coumaric acid, cinnamaldehyde, and thymoquinone showed moderate binding affinities to HSPA5 SBDβ. Phytoestrogens bear the same recognition affinity to HSPA5 SBDβ. Estrogenic hormones such as estrogens, progesterone, testosterone, and cholesterol have also binding affinities to HSPA5 SBDβ. From the binding affinity, phytoestrogens and estrogens are found to be the most feasible ligands to bind to HSPA5. Phytoestrogens such as daidzein, genistein, formononetin, and biochanin A also bind to estrogen receptors (ER) of humans and murines *in silico* and act like estrogen-like molecules (Sayed and Elfiky, 2018). Olive leaf hydroxytyrosol has moderate binding affinity to HSPA5 SBDβ. CA and *p*-coumaric acid also have average binding affinities to surface HSPA5 SBDβ and compete for recognition by the S-glycoprotein. The CAPE has a medium binding affinity to HSPA5 SBDβ. Cinnamaldehyde and thymoquinone have average binding affinity to HSPA5 SBDβ.

### Inhibition of SARS-CoV by Plant Lectins

Plant lectins are potent inhibitors of CoV infection of host cells. Plant lectin-like protein interacts with virus surface proteins. Agglutinins, including mannose-specific lectin, inhibit the attachment and replication of SARS-CoV. Lectins can inhibit SARS-CoV-2 infection. Lectins such as griffithsin exert anti-CoV activity by multiple mechanisms (Moghaddam et al., 2014; Dai et al., 2019). Of the 33 plant lectins screened for the inhibition of SARS-CoV, *L. radiata* agglutinin was found to be effective (Keyaerts et al., 2007). GlcNAc-specific lectin, (GlcNAc)n-specific lectin, Gal-specific lectin, Man/Glc-specific lectin, Gal/GalNAc-specific lectin, GalNAcα(1.3)Gal > GalNAc > Gal-specific lectin, and Man/GalNAc-specific lectins inhibit the viral attachment to host cells and replication in host cells (Keyaerts et al., 2007). For example, *Urtica dioica* L. agglutinin inhibits viral replication in the penetration stages and binds to the S-glycoprotein and GlcNAc-like residues on the envelope glycan (Kumaki et al., 2011). Lectins from *Allium porrum* L., *Nicotiana tabacum* L., and *U. dioica* inhibit the virus propagation (EC50) (Yonesi and Rezazadeh, 2020).

Plant lectins are promising antiviral agents against influenza, herpes simplex virus (Hwang et al., 2020), and Ebola (Michelow et al., 2011; Covés-Datson et al., 2019). *Galanthus nivalis* L. agglutinin recognizes the S-glycoprotein and membrane proteins of feline CoV. Red alga *Griffithsia* sp. griffithsin directly interacts with S-glycoprotein (O’Keefe et al., 2010) and MERS-CoV (Millet et al., 2016). Griffithsin lectin purified from the *Griffithsia* sp. has three identical glycan-binding domains (GBDs) (O’Keefe et al., 2010). Different inhibition spectrums of griffithsin against different strains may be caused by genomic differences of the S-glycoproteins between SARS-CoV strains, potentiating different binding to the GBDs and affinity to the S-glycoproteins. Griffithsin lectin is relatively a small molecule and classified to be a MERS-CoV and HCoV inhibitor (EC50 of 0.0032–0.33 µM) (Millet et al., 2016). The three carbohydrate-binding domains specific for S-glycoprotein glycans inhibit MERS-CoV viral attachment to host cells (EC50 of 0.125 µM) (O’Keefe et al., 2010). Griffithsin has low toxicity and is a candidate agent against SARS-CoV-2. Griffithsin was effective for the SARS-CoV Urbani/Tor-II strains and not effective for the Frank strain. Human mannose-binding lectin (MBL) protects mice from fatal Ebola infections (Michelow et al., 2011). The legume Jack bean, *Canavalia ensiformis* (L.) DC., lectin concanavalin A (Con-A) is a phytagglutinin that hemagglutinates the hemagglutinating encephalomyelitis CoV, via binding to glycoconjugates (Greig and Bouillant, 1977). The therapeutic utility of Con-A is limited due to its hepatotoxic side effects. Leguminous *Dioclea lasiocarpa* Mart. ex Benth. lectin DLasiL inhibits feline CoV at an EC50 of 5 nM. Interestingly, *Galanthus nivalis* L. lectin, agglutinin, recognizes the S-glycoprotein and feline coronavirus (FCoV) NTU156 (Hsieh et al., 2010). *Griffithsia* sp. griffithsin blocks PEDV (NJPEDV) attachment to host cells (Li et al., 2019).

### Virus Entry Inhibitors via Nonspecific Inhibition

Lycorine, emetine, berbamine, and mycophenolate mofetil are known to inhibit several CoV strains including MHV-A59, HCoV-OC43/-NL63, and MERS-CoV (Shen et al., 2019). Additionally, mycophenolate mofetil showed immunosuppressive activity on the related virus-infected cells. Similarly, marine brown alga species *Ecklonia cava* Kjellman eckols, 7-phloroeckol, phlorofucofuroeckoln, and dieckol, blocked virus binding to porcine epidemic cells (Kwon et al., 2013). *Cinnamomum cassia* (L.) J. Presl. cortex procyanidin A2/B1 and cinnamtannin B1 inhibited SARS-CoV infection (Zhuang et al., 2009). Among these, procyanidin A2 inhibits the early stage of virus entry by blocking the clathrin-dependent endocytosis pathway. As virus entry inhibitors, tetra-O-galloyl-beta-d-glucose and luteolin prevent SARS-CoV entry into host cells (Yi et al., 2004). Upon interaction with ACE2, SARS-CoVs are incorporated into vesicle forms to facilitate entry into the cells. Juglanin inhibits SARS-CoV channel 3a (Schwarz et al., 2014). (−)-Catechin gallate and (−)-gallocatechin gallate block the nanoparticle-based RNA oligomer of SARS-CoV (Roh, 2012). *Houttuynia cordata* Thunb. quercetin, quercetrin, rutin, and cinanserin inhibit murine CoV (Chiow et al., 2016).

*Aglaia foveolata* Pannell sivestrol blocks Cap-dependent translation of HCoV-229E mRNA genome (Müller et al., 2018). Ouabain reduces the viral titers, yields, and viral RNA copy numbers (Yang et al., 2018). Its carboxylic amide derivatives exhibit specific SARS-CoV antiviral activity (Kim et al., 2019). Plant alkaloids such as cepharanthine, tetrandrine, and fangchinoline protected HCoV-OC43–infected human lung MRC-5 cells from cell death (Kim et al., 2019; Majnooni et al., 2020). Cepharanthine also blocks the SARS-CoV protease enzyme (Zhang et al., 2005). Some diterpenes, sesquiterpenes, triterpenes, lignans, and curcumin also exhibited antiviral activities against SARS-CoV (Yamagata et al., 2019). The marine algae *Halimeda tuna* (Ulvophyceae, Chlorophyta) diterpene aldehyde, halituna, shows antiviral activity against murine CoV A59 (Koehn et al., 1991). Some compounds inhibit SARS-CoV S-protein RBD interaction with ACE2. For example, the cathepsin L inhibitor inhibits fusion of viral membrane with host cell PM, blocking virus entry (Adedeji et al., 2013). On the other hand, *Aglaia* sp. silvestrol specifically inhibits the RNA helicase eIF4A of MERS-CoV (Müller et al., 2018). The *Boenninghausenia sessilicarpa* H. Lév. bioactive coumarin, leptodactylone, exhibits cytopathogenic effects on SARS-CoV–infected cells (Yang et al., 2007). *Pelargonium sidoides* DC. 11% ethanol extract interferes with the virus surface and causes inactivation of respiratory viruses (Michaelis et al., 2011).

For animal CoVs, *Sambucus nigra* L. lectins and flavonols also disrupt virion structure, compromising virus membrane integrity of avian IBV (Chen et al., 2014). *Mentha piperita* L., *Thymus vulgaris* L., and *Desmodium canadense* (L.) DC. 40% ethanol extracts directly inactivate the virus envelope structure of avian IBV (Lelesius et al., 2019). *Houttuynia cordata* essential oils and methyl-nonyl-ketone inhibit the release of avian IBV (Yin et al., 2011). Plant eucalyptol blocks the interaction of RNA with the nucleocapsid protein of avian IBV (Yang et al., 2010), and α-/β-pinene suppresses the N-protein function, hindering the interaction of avian IBV RNA and N-protein (Yang et al., 2011). *Forsythia suspensa* (Thunb.) Vahl forsythoside A affects cell signaling of avian IBV-infected avian cells (Li et al., 2011). For bovine CoVs, *Rosa nutkana* C. Presl and *Amelanchier alnifolia* (Nutt.) Nutt. ex M. Roem. prunasin exhibits cytotoxicity against BCoV (McCutcheon et al., 1995). *Ziziphus jujuba* Mill. jubanine G and H as well as nummularine B exhibit cytotoxicity in PEDV-infected cells (Kang et al., 2015). *Ginkgo biloba* polysaccharides dose-dependently inhibit viral attachment and the entry steps of PEDV CoV-777 (Lee et al., 2015). *Houttuynia cordata* quercetin 7-rhamnoside interacts directly with PEDV (Song et al., 2011). *H. cordata* quercetin 7-rhamnoside, quercetin, apigenin, and luteolin exhibit cytotoxicity in PEDV-infected host cells (Choi et al., 2009). *Prunus serrulata* var. *spontanea* (Maxim.) E.H. Wilson polyphenols exhibit cytotoxicity in PEDV (KPEDV9)-infected host cells (Yook et al., 2010).

### Carcinoembryonic Antigen Cell Adhesion Molecule Receptor

The N-terminal domain of S1 recognizes CEACAM1. S-glycoprotein–CEACAM receptor binding leads to S-glycoprotein–mediated fusion of membrane. For example, MHV recognizes the CEACAM expressed on BHK cell cultures (Heino et al., 2000). In MERS-CoV, CEACAM5 isoforms are associated with attachment (Naskalska et al., 2019). Therefore, MERS-CoV recognizes CEACAM5 as the attachment and entry site (Chan et al., 2016). In the structural aspect, the S1 N-terminal domain exhibits an identical tertiary structure compared with human galectins which recognize Gal-residues. The S1 N-terminal domain of the MHV recognizes mouse CEACAM1a and that of BCoV recognizes carbohydrate residues (Peng et al., 2011; Peng et al., 2012; Walls et al., 2016). Because CEACAM1a mRNA is alternatively spliced, HCoVs have been suggested to be evolutionarily recombinant between the host galectin and the S1-glycoprotein genes. However, the BCoV S1-glycoprotein gene is not subjected to such recombination but bears the glycan-binding lectin activity. MHV S1-glycoprotein has also been suggested to acquire mouse CEACAM1a-binding capacity (Peng et al., 2017), suggesting that CoVs receive evolutionary pressure to acquire the interaction capacity with host receptors over cross-species (Li, 2015; Li, 2016). Moreover, soluble forms of CEACAM directly involve in S-glycoprotein–mediated PM fusion, inducing conformational shifts (Matsuyama and Taguchi, 2002; Taguchi and Matsuyama, 2002). On the host side, host organisms have also evolved to escape the lethal pressure from coronavirus infections. The acquired geno- and phenotypes of such hosts are expressed for SA-recognizing proteins. For example, Siglecs are representatively expressed to utilize the innate responses of host immune cells.

### Major Histocompatibility Complex Class I (MHC-I) C and DC-SIGN (CD209) for Coronavirus Attachment Site

CoV-HKU1 spikes additionally bind to MHC-I C (Chan et al., 2009). HCoV-HKU1 S-glycoprotein also binds to MHC-IC known as HLA-C (Song et al., 2011). SARS-CoV utilizes dendritic cell (DC)–specific intercellular adhesion molecule (ICAM)-3–grabbing nonintegrin (DC-SIGN) (Marzi et al., 2004). SARS-CoV also uses the C-type lectins of DC-SIGN and DC-L-SIGN. DC/L-SIGN recognizes the S-glycoprotein glycans, where seven N-glycan sites are known to enable DC/L-SIGN–mediated infection (Marzi et al., 2004; Han et al., 2007).

### Dipeptidyl Peptidase-4, Aminopeptidase N, and Tetraspanin CD9

Ser exopeptidase dipeptidyl peptidase-4 (DPP-4)/human CD26 is the MERS-CoV receptor. DPP4 is a ubiquitous membrane-type aminopeptidase in the PM. The MERS-CoV S1 N-terminal domain binds to DPP4 (Raj et al., 2013; Gheblawi et al., 2020; Letko et al., 2020). The CD9 tetraspanin, but not the CD81 tetraspanin, interacts with DPP4 and TMPRSS2 (Earnest et al., 2017). These CD9–DPP4–TMPRSS2 receptors and proteases permit entrance of the MERS-CoV pseudovirus into the host cells. Tetraspanin CD9 binds to the DPP4–TMPRSS2 complex, and this triggers the S-glycoprotein. The *α*-CoV HCoV-229E S-glycoprotein binds to human aminopeptidase N (hAPN) (Yeager et al., 1992). hAPN (CD13) is a TM alanyl aminopeptidase and Zn-dependent metalloprotease (EC 3.4.11.2) with a MW of 150 kDa and made up of 967 amino acids. The C-terminal domain has zinc–MMP-related pentapeptides. The S1 C-terminal region is the APN-binding domain (Deng et al., 2016). Porcine APN (pAPN) and hAPN exhibit about 80% protein similarity. PEDV can bind to hAPN and neuraminic acid as its co-receptors, as human cytomegalovirus, pCoV, FIPV, feline enteric virus (FeCV), and canine CoVs recognize them (Delmas et al., 1992; Söderberg et al., 1993; Tresnan et al., 1996; Nomura et al., 2004). APN is the functional receptor for HCoV-229E (Yeager et al., 1992; Zhu et al., 2018). Bestatin, an APN inhibitor, binds to its catalytic site (Milewska et al., 2014).

### Heparan Sulfate as Human Coronavirus Entry Site

MHV and HCoV-NL63 are known to interact with heparan sulfate (HS) (Watanabe et al., 2007; Milewska et al., 2014). The HS proteoglycans (HSPGs) are recognized by M-protein in the absence of the S-glycoprotein in the HCoV-NL63 entry into host cells. Then, the M-protein and S-glycoprotein enhance virus entry into the host cells (Milewska et al., 2014; Naskalska et al., 2019). In general, ACE2, APN, HSPA5, furin, O-Ac-neuraminic acid, and HSPGs are the CoV-binding molecules. Apart from the precise targeting of the molecules, several medicinal plant resources also exhibit antiviral activities against respiratory and influenza virus diseases. For example, *Panax ginseng* can prevent viral respiratory diseases and influenza virus diseases (Cheng et al., 2020). *Pelargonium sidoides* also prevents respiratory viral infections (Im et al., 2015). *Astragalus mongholicus* Bunge can treat common cold and upper respiratory infections and also prevent influenza virus infections (Kolodziej, 2011; Liang et al., 2019). Compounds and extracts with anti-CoV activities are summarized in Table 1.

**TABLE 1:** Summary of anti-CoV compounds and extracts.

**Names of compounds and extracts**	**Target specificity**	**Plant**	**References**
Lycorine	Cytopathogenic	*Lycoris radiata* (L’Hér.)	Yu et al. (2012)
Silvestrol	RNA helicase eif4a	*Aglaia* sp.	Miean and Mohamed, (2001), Lau et al. (2008)
Tryptanthrin	RdRp, PLpro 2	*S. cusia* (Nees) Kuntze	Wen et al. (2007)
Saikosaponin	Replication/entrance, S-glycoprotein	*Bupleurum* sp., *Heteromorpha* sp., *Scrophularia scorodonia* L.	Ulasli et al. (2014), Ushio and Abe, (1992), Chiang et al. (2003)
Cafffeic acid (chlorogenic acid, isoferulic acid), CAPE	Mpro, GRP78 (HSPA5)	*Sambucus javanica* subsp. *chinensis* Fukuoka (elderberry)	Li. (2015), Weng et al. (2019), Wang et al. (2009), Kumar et al. (2020), Sayed and Elfiky (2018)
Linolenic acid, palmitic acid, oleanolic acid	3CLpro, Mpro	*Allium cepa* L.	Cheng et al. (2006), Sharma and Kaur (2020), Fitriani et al. (2020)
Isobavachalcone, broussochalcone A/B	3CLpro, PLpro	*Broussonetia papyrifera* (L.) L'Hér. Ex vent	Ryu et al. (2010)
Myricetin	Helicase nsp13, NLRP3 inflammasome, 3CLpro	*Isatis indigotica* Fort., *Torreya nucifera*, *Vaccinium oxycoccos* L., *Calamus scipionum* Lam.	Cho et al. (2013), Mikulic-Petkovsek et al. (2012), Qing et al. (2016), Yang et al. (2010), Chen et al. (2019)
Psoralidin	PLpro	*Psoralea corylifolia* L.	Kim et al. (2014)
Quercetin	Plpro, 3CLpro, NLRP3	*Psoralea corylifolia* L.	Ryu et al. (2010), Kim et al. (2014)
Scutellarein	Helicase nsp13, ATPase, 3CLpro	*Isatis indigotica* Fort., *Torreya nucifera*, *S. baicalensis*	Cho et al. (2013), Yang et al. (2010)
Silvestrol	NLRP3 inflammasome, RNA helicase eIF4A	*Aglaia* sp.	Lim et al. (2018), Müller et al. (2018)
Baicalin	NLRP3 inflammasome, S-glycoprotein, Nsp9, replication	*Scutellaria baicalensis*, *S. lateriflora*	O’Flaherty et al. (2019), Chandel et al. (2020), Lim et al. (2018), Fu et al. (2016)
Wogonoside	NLRP3 inflammasome	*Scutellaria baicalensis*	Lim et al. (2018), Sun et al. (2015)
Kaempferol	NLRP3 inflammasome, PLpro, 3CLpro, 3a channel protein	*Rosmarinus officinalis*, *Sambucus nigra*, *Viola odorata* L.	Lim et al. (2018), Khaerunnisa et al. (2020), Schwarz et al. (2014)
Apigenin, flavones, biflavones, amentoflavone, bilobetin, ginkgetin, sciadopitysin	NLRP3 inflammasome, 3CLpro	*Torreya nucifera* (L.) Siebold & Zucc., *Ginkgo biloba* L., *Chamaecyparis obtusa* (Siebold & Zucc.) Endl., *Hypericum perforatum* L.	Lim et al. (2018), Yamagata et al. (2019), Ryu et al. (2010)
Tomentin (3′-O-/4′-O-methyldiplacol, 3′-O-/4′-O-methyldiplacone, mimulone, diplacone, 6-geranyl-4′,5.7-trihydroxy-3′,5′-dimethoxyflavanone)	PLpro	*Paulownia tomentosa* (Thunb.) Steud.	Lung et al. (2020)
Tryptanthrin	PLpro-2	*Strobilanthes cusia* (Nees) Kuntze	Wen et al. (2007), Chen et al. (2008)
Indigodole B	PLpro-2	*Strobilanthes cusia* (Nees) Kuntze	Wen et al. (2007)
Theaflavin	RdRp	*Camellia sinensis* (L.) Kuntze	Zhang et al. (2020)
3,4,5-Trihydroxy-1,8-bis [(2 R,3 R)-3,5,7-trihydroxy-2-chromanyl]-6-benzo()annulenone	RdRp	*Camellia sinensis* (L.) Kuntze	Zhang et al. (2020)
Betulonic acid	Replication, 3CLpro	*Sequoia sempervirens* (D.Don) Endl.	Loizzo et al. (2008)
Betulinic acid	Replication, 3CLpro	*Sequoia sempervirens* (D.Don) Endl.	Loizzo et al. (2008).Cheng et al. (2006)
8β-hydroxyabieta-9 (11),13-dien-12-one, 3β,12-diacetoxyabieta-6,8,11,13-tetraene	Replication	*Sequoia sempervirens* (D.Don) Endl.	Loizzo et al. (2008)
Curcumin	Replication, Mpro	*Curcuma longa*	Loizzo et al. (2008), Yamagata et al. (2019), Khaerunnisa et al. (2020)
Hinokinin	Replication	*Sequoia sempervirens* (D.Don) Endl.	Loizzo et al. (2008)
Savinin	Replication, 3CLpro	*Sequoia sempervirens* (D.Don) Endl.	Loizzo et al. (2008), Islam et al. (2020)
Glycyrrhizin, 18β-glycyrrhetinic acid, licochalcone	S-glycoprotein, N-protein, attachment/entry	*Glycyrrhiza uralensis* Fisch. (licorice), *G. glabra* L., *G. inflata* Bat.	Hoever et al. (2005), Shen et al. (2019), Haiying et al. (2003), Fu et al. (2016), Cinatl et al. (2003), Michaelis et al. (2011)
Vitamins A and C	Host immunity	Various	Häkkinen et al. (1999)
Lignin	Replication, 3CLpro	*Sequoia sempervirens* (D.Don) Endl.	Cheng et al. (2006), Yamagata et al. (2019), Islam et al. (2020), Park et al. (2012)
Desmethoxyreserpine	Replication, 3CLpro	*Rauvolfia canescens*	Cheng et al. (2006)
Β-Ocimene	Replication	*Laurus nobilis* L.	McDonagh et al. (2014)
1,8-Cineole	Replication, 3CLpro, N-protein	*Laurus nobilis* L.	Müller et al. (2020), McDonagh et al. (2014), Yang et al. (2011), Yang et al. (2010)
*α*-Pinene	Replication, N-protein	*Laurus nobilis* L., *Rosmarinus officinalis* L. (rosemary)	Müller et al. (2020), McDonagh et al. (2014), Yang et al. (2011)
β-Pinene	Replication, N-protein	*Laurus nobilis* L.	Müller et al. (2020), McDonagh et al. (2014), Yang et al. (2011)
δ3-Carene	Replication	*Laurus nobilis* L.	McDonagh et al. (2014)
*α*-Cedrol	Replication	*Laurus nobilis* L.	McDonagh et al. (2014), Kim et al. (2008)
Blancoxanthone	Cytopathic effects	*Calophyllum blancoi* Planch. & Triana	Kim et al. (2010)
Pyranojacareubin	Cytopathic effects	*Calophyllum blancoi* Planch. & Triana	Kim et al. (2010)
Gallic acid	S-glycoprotein	Tea leaves, oak bark	Wang et al. (2009)
Artemisinin	Replication	*Artemisia annua* L.	O’Flaherty et al. (2019), de Vries et al. (1997)
Quercetin, quercetin 7-rhamnoside, quercetrin	Replication, NLRP3 inflammasome, 3CLpro, PLpro	*Torreya nucifera* (L.) Siebold & Zucc., *Houttuynia cordata* Thunb.	O’Flaherty et al. (2019), Choe and Kim, (2017), Ryu et al. (2010), Theerawatanasirikul et al. (2020), Chiow et al. (2016), Lee et al. (2015), Song et al. (2011)
Rutin	Replication	*Houttuynia cordata* Thunb.	Li et al. (2005), Packer and Cadenas, (2011), O'Flaherty et al. (2019)
Hesperidin, hesperitin	Replication Mpro, ACE2, RdRp	*Citrus aurantium* L.	O'Flaherty et al. (2019), Rasool et al. (2020)
Glycyrrhizic acid, glycyrrhetinic acid, glycyrrhizin	Replication, neucleoprotein, S-glycoprotein	*Glycyrrhiza uralensis* Fisch. (licorice), *G. glabra* L., *G. inflata* Bat.	Li et al. (2005), Packer and Cadenas (2011), O'Flaherty et al. (2019), Fu et al. (2016)
Ferulic acid (caffeoyltyramine, feruloyltyramine, feruloyloctopamine)	Replication, PLpro	*Cimicifuga racemosa* L., *Melia* sp., *Coptis* sp., *Phellodendron* sp., *Sophora subprostrata* Chun & T.Chen. (Fabaceae)	Song et al. (2014), Jin et al. (2019), Sun et al. (2019), Li. (2015)
Isoferulic acid	Replication, PLpro	*Cimicifuga racemosa* L., *Melia* sp., *Coptis* sp., *Phellodendron* sp., *Sophora subprostrata* Chun & T.Chen. (Fabaceae)	Song et al. (2014),Jin et al. (2019),Sun et al. (2019)
Toosendanin	Replication, RdRp	*Cimicifuga racemosa* L., *Melia* sp., *Coptis* sp., *Phellodendron* sp., *Sophora subprostrata* Chun & T.Chen. (Fabaceae)	Song et al. (2014), Jin et al. (2019), Sun et al. (2019)
Berberine	Replication, RdRp	*Cimicifuga racemosa* L., *Melia* sp., *Coptis* sp., *Phellodendron* sp., *Sophora subprostrata* Chun & T.Chen. (Fabaceae)	Song et al. (2014), Jin et al. (2019), Sun et al. (2019)
Matrine	Replication, RdRp	*Cimicifuga racemosa* L., *Melia* sp., *Coptis* sp., *Phellodendron* sp., *Sophora subprostrata* Chun & T.Chen. (Fabaceae)	Song et al. (2014),Jin et al. (2019),Sun et al. (2019)
Oxymatrine	Replication, RdRp	*Cimicifuga racemosa* L., *Melia* sp., *Coptis* sp., *Phellodendron* sp., *Sophora subprostrata* Chun & T.Chen. (Fabaceae)	Song et al. (2014), Jin et al. (2019), Sun et al. (2019)
Sophoranone	Replication, RdRp	*Cimicifuga racemosa* L., *Melia* sp., *Coptis* sp., *Phellodendron* sp., *Sophora subprostrata* Chun & T.Chen. (Fabaceae)	Song et al. (2014), Jin et al. (2019), Sun et al. (2019)
Sophocarpine	Replication, RdRp	*Cimicifuga racemosa* L., *Melia* sp., *Coptis* sp., *Phellodendron* sp., *Sophora subprostrata* Chun & T.Chen. (Fabaceae)	Song et al. (2014), Jin et al. (2019), Sun et al. (2019)
Khainaosides	S-glycoprotein, Nsp15, Mpro	*Vitex glabrata *	Adem et al. (2021)
6-O-caffeoylarbutin	S-glycoprotein, Nsp15, Mpro	*Vaccinium dunalianumas*	Adem et al. (2021)
Vitexfolin	S-glycoprotein, Nsp15, Mpro	*Vitex rotundifolia*	Adem et al. (2021)
Toosendanin	RNA polymerase complex	*Melia azedarach* L.	Simmons et al. (2013)
Matrine	Myd88/NF-κB, NLRP3 inflammasome	*Sophora flavescens* Aiton	Hulswit et al. (2019)
Tetrandrine	Replication, S-glycoprotein, N-protein	*Stephania tetrandra* var. *glabra*	Kim et al. (2019)
Fangchinoline	Replication, S-glycoprotein, N-protein	*Stephania tetrandra* var. *glabra*	Kim et al. (2019)
Cepharanthine	Replication, S-glycoprotein, N-protein	*Stephania tetrandra* var. *glabra*	Kim et al. (2019)
Actinomycin D	Cell attachment	*Streptomyces parvulus*	O'Flaherty et al. (2019)
Ginsenoside (gynosaponin)	Cell attachment, S-glycoprotein	*Panax ginseng* (T.Nees) C.A.Mey	Wu et al. (2020)
Resveratrol	Replication, N-protein	*Vitis vinifera*, *Polygonum cuspidatum*, *Vaccinium macrocarpon*	Lin et al. (2005)
Omacetaxine (homoharringtonine)	Replication, N-protein, S-glycoprotein	*Tylophora indica* (Burm.f.) Merr.	Cao et al. (2015)
Tylophorine, 7-methoxycryptopleurine	Replication, N- and S-glycoprotein, NF-κB, JAK	*T. indica*	Yang et al. (2010), Cho et al. (2006), Yang et al. (2017)
Dihydrotanshinone, tanshinone, cryptotanshinone, rosmariquinone	S-glycoprotein, 3CLpro, PLpro	*Salvia miltiorrhiza* Bunge	Zhang et al. (2020), Park et al. (2012)
Tetra-O-galloyl-β-d-glucose	S2-protein	*Rhus chinensis* Mill.	Yi et al. (2004)
Luteolin, 7-methylluteolin, 7-benzyl luteolin	S-glycoprotein, NLRP3 inflammasome, 3CLpro, cytotoxicity	*Rhus chinensis* Mill., *Torreya nucifera* (L.) Siebold & Zucc., *Houttuynia cordata*	Yi et al. (2004), Zhang et al. (2018), Ryu et al. (2010), Theerawatanasirikul et al. (2020), Song et al. (2011), Choi et al. (2009)
Alstotide	M-protein, S-glycoprotein	*Alstonia scholaris* (L.) R.Br.	Nguyen et al. (2015)
Polyphenols	S-glycoprotein	*Punica granatum* L.	Sundararajan et al. (2010)
Steviol	3CLpro	*Stevia rebaudiana*	Theerawatanasirikul et al. (2020), Theerawatanasirikul et al. (2020)
Phyllaemblicin	ACE2	*Phyllanthus emblica* L.	Meneguzzo et al. (2020)
Neohesperidin, hesperidin	ACE2	*Citrus aurantium* L.	Meneguzzo et al. (2020)
Chrysin	ACE2	*Oroxylum indicum*	Basu et al. (2020)
Emodin	S-glycoprotein, 3CLpro	*Isatis indigotica* Fortune ex Lindl.	Ho et al. (2007), Islam et al. (2020), Lin et al. (2005)
Hesperetin	3CLpro	*Isatis indigotica*	Islam et al. (2020)
Anthraquinone	3CLpro	*Rheum palmatum* L.	Luo et al. (2009)
Cadambine	3CLpro	*Uncaria tomentosa* (Willd. ex Schult.) DC.	Yepes-Pérez et al. (2020)
Speciophylline	3CLpro	*Uncaria tomentosa* (Willd. ex Schult.) DC.	Yepes-Pérez et al. (2020)
Proanthocyanidin	3CLpro	*Uncaria tomentosa* (Willd. ex Schult.) DC.	Yepes-Pérez et al. (2020)
Silymarin	Adam17	*Silybum marianum* (L.) Gaertn., *Cynara cardunculus* L., *Curcuma longa* L.	Borah et al. (2016)
Lycorine, berbamine	Viral entry, RNA, DNA, and protein synthesis	*Lycoris radiata* (Amaryllidaceae), *Berberis amurensis*	Shen et al. (2019)
Mycophenolate	Immunosuppression	*Penicillium stoloniferum*	Shen et al. (2019)
Eckol, 7-phloroeckol, phlorofucofuroeckoln, dieckol	Attachment, entry, replication, S-glycoprotein	Brown alga *Ecklonia cava* Kjellman	Kwon et al. (2013)
Procyanidin A2/B1, cinnamtannin B1	Virus entry, transferrin receptor	*Cinnamomum cassia* (L.) J. Presl	Zhuang et al. (2009)
Tetra‐O‐galloyl‐beta‐d‐glucose, luteolin	Virus entry, S-glycoprotein	*Galla chinensis*, *Veronica linariifolia* Pall.	Yi et al. (2004)
Juglanin	Channel 3a	*Quercus ilex* L., Viola odorata L.	Schwarz et al. (2014)
Catechin gallate, gallocatechin gallate	N-protein, RNA oligomer	Green tea, buckwheat, *Dianthus caryophyllus*	Roh. (2012)
Cinanserin	Virus entry, replication	*Houttuynia cordata* Thunb.	Chiow et al. (2016)
Sivestrol	Cap-dependent translation	*Aglaia foveolata* Pannell	Müller et al. (2018)
Ouabain	Replication, cell membrane sodium/potassium pump, na^+^/k^+^-ATPase	*Acokanthera schimperi, Strophanthus gratus*	Yang et al. (2018)
Cepharanthine	Protease, cytotoxicity, S- and N-protein expression	*Stephania* *tetrandra*, Menispermaceae species	Kim et al. (2019), Zhang et al. (2005)
Fangchinoline	Cytotoxicity, S- and N-protein expression	*Stephania* *tetrandra*, Menispermaceae species	Kim et al. (2019)
Tetrandrine	Cytotocixity, S- and N-protein expression	*Stephania* *tetrandra*, Menispermaceae species	Kim et al. (2019)
Diterpene aldehyde, halituna	Cytotoxicity	Marine algae *Halimeda tuna* (Ulvophyceae, Chlorophyta)	Yamagata et al. (2019), Koehn et al. (1991)
Coumarin	Cytotoxicity	*Boenninghausenia sessilicarpa* H.Lév.	Yang et al. (2007)
Leptodactylone	Cytotoxicity	*Boenninghausenia sessilicarpa* H.Lév.	Yang et al. (2007)
Methyl-nonyl-ketone	Virus release	*Houttuynia cordata*	Yin et al. (2011)
Eucalyptol	RNA with N-protein	*Eucalyptus globulus* oil	Yang et al. (2010)
α-/β-Pinene	N-protein	*Sideritis* spp., *Salvia* spp., *Cannabis*	Yang et al. (2011)
Forsythoside A	Signaling, replication	*Forsythia suspensa* (Thunb.) Vahl	Li et al. (2011)
Prunasin	Cytotoxicity	*Rosa nutkana* C. Presl, *Amelanchier alnifolia* (Nutt.) Nutt. ex M.Roem.	McCutcheon et al. (1995)
Jubanine G/H	Cytotoxicity	*Ziziphus jujuba* Mill.	Kang et al. (2015)
Nummularine B	Cytotoxicity	*Ziziphus jujuba* Mill.	Kang et al. (2015)
Polysaccharide	Viral attachment	*Ginkgo biloba*	Lee et al. (2015)
Polyphenol	Cytotoxicity	*Prunus serrulata* var. *spontanea* (Maxim.) E.H. Wilson	Yook et al. (2010)
Afzelin (kaempferol rhamnoside)	Mpro, ACE2, RdRp	*Nymphaea odorata*	Rasool et al. (2020)
Biorobin	Mpro, ACE2, RdRp	*Acalypha indica*	Rasool et al. (2020)
δ-Viniferin (resveratrol dehydrodimer)	Mpro, ACE2, RdRp	*Vitis vinifera*	Rasool et al. (2020)
Taiwanhomoflavone A	Mpro, ACE2, RdRp	*Cephalotaxus wilsoniana*	Rasool et al. (2020)
Lactucopicrin 15-oxalate	Mpro, ACE2, RdRp	Asteraceae	Rasool et al. (2020)
Nympholide A	Mpro, ACE2, RdRp	*Nymphaea lotus* Linn.	Rasool et al. (2020)
Phyllaemblicin B	Mpro, ACE2, RdRp	*Phyllanthus emblica*	Rasool et al. (2020)
5,7,3′,4′-Tetrahydroxy-2’-(3,3-dimethylallyl) isoflavone	Mpro	*Psorothamnus arborescens* var. *simplifolius* (Parish) Barneby	Joshi et al. (2020)
Myricitrin	Mpro	*Myrica cerifera* L.	Joshi et al. (2020)
Methyl rosmarinate	Mpro	*Myrica cerifera* L.	Joshi et al. (2020)
Withaferin	Mpro	*Ashwagandha* species	Kumar et al. (2020)
Oleuropein	Mpro	*Olea europaea*	Khaerunnisa et al. (2020)
Ermanin	Mpro	*Tanacetum microphyllum*	Erlina et al. (2020)
Jensenone	Mpro	*Eucalyptus jensenii*	Sharma and Kaur, (2020)
Oleanolic acid	Mpro	*Allium cepa* L.	Fitriani et al. (2020)
α-Tocotrienol	Mpro	*Cocos nucifera* L.	Fitriani et al. (2020)
Asiatic acid	Mpro	*Psidium guajava* L.	Fitriani et al. (2020)
Culinoside	Mpro	*Eucalyptus globulus*	Fitriani et al. (2020)
Cinnamic amide	PLpro	*Tribulus terrestris* L.	Song et al. (2014)
Tannin, tannic acid	3CLpro	*Rhus semialata*, *Quercus infectoria*, *Rhus coriaria*	Islam et al. (2020), Chen et al., (2005)
Coumarin	3CLpro	*Cinnamomum cassia*, *Dipteryx odorata*	Islam et al. (2020)
8β-Hydroxyabieta-9 (11),13-dien-12-one	3CLpro	*Thuja standishii* (Cupressaceae)	Islam et al. (2020)
Stictic acid	3CLpro	*Rheum palmatum* L.	Theerawatanasirikul et al. (2020)
Coumaroyltyramine	3CLpro, PLpro	*Allium fistulosum*	Cheng et al. (2006)
Moupinamide (feruloyltyramine)	3CLpro, PLpro	*Piper nigrum*	Cheng et al. (2006)
Dihomo-c-linolenic acid	3CLpro, PLpro	*Asphodelus tenuifolius*, *Aizoon canariense*, *Emex spinosus*	Cheng et al. (2006)
Sugiol	3CLpro, PLpro	*Calocedrus formosana* Florin (Cupressaceae)	Cheng et al. (2006)
Tanshinone, dihydrotanshinone, methyl tanshinonate, cryptotanshinone, rosmariquinone, N-cis-feruloyltyramine	3CLpro, PLpro, androgen receptor	*Salvia miltiorrhiza* Bunge	Cheng et al. (2006), Park et al. (2012), Xu et al. (2012)
3-Isotheaflavin-3-gallate	3CLpro	Green tea, black tea, puer tea	Chen et al. (2005)
Theaflavin-3,3′-digallate	3CLpro	Green tea, black tea, puer tea	Chen et al. (2005)
Sinigrin	3CLpro	*Isatis indigotica* Fortune ex Lindl.	Islam et al. (2020), Lin et al. (2005)
Indigo	3CLpro	*Isatis indigotica* Fortune ex Lindl.	Islam et al. (2020), Lin et al. (2005)
Hesperetin	3CLpro	*Isatis indigotica* Fortune ex Lindl.	Lin et al. (2005)
Β-sitosterol	3CLpro	*Isatis indigotica* Fortune ex Lindl.	Islam et al. (2020), Lin et al. (2005)
Kazinol A/B/F/J	3CLpro, PLpro	*B. papyrifera*	Ryu et al. (2010)
Broussoflavan A	3CLpro, PLpro	*B. papyrifera*	Ryu et al. (2010)
4-Hydroxyisolonchocarpin	3CLpro, PLpro	*B. papyrifera*	Ryu et al. (2010)
Papyriflavonol	3CLpro, PLpro	*B. papyrifera*	Ryu et al. (2010)
3′-(3-Methylbut-2-enyl)-3′,4.7-trihydroxyflavane	3CLpro, PLpro	*B. papyrifera*	Ryu et al. (2010)
Papyriflavonol A	PLpro	*B. papyrifera*	Ryu et al. (2010)
Bavachinin	PLpro	*Psoralea corylifolia* L.	Lin et al. (2005), Kim et al. (2014)
Neobavaisoflavone	PLpro	*Psoralea corylifolia* L.	Lin et al. (2005), Kim et al. (2014)
Isobavachalcone	PLpro	*Psoralea corylifolia* L.	Lin et al. (2005), Kim et al. (2014)
4′-O-methylbavachalcone	PLpro	*Psoralea corylifolia* L.	Lin et al. (2005), Kim et al. (2014)
Corylifol A	PLpro	*Psoralea corylifolia* L.	Lin et al. (2005), Kim et al. (2014)
Psoralidin	PLpro	*Psoralea corylifolia* L.	Lin et al. (2005), Kim et al. (2014)
Celastrol	3CLpro	*Triterygium regelii* Sprague & Takeda	Ryu et al. (2010)
Pristimerin	3CLpro	*Triterygium regelii* Sprague & Takeda	Ryu et al. (2010)
Tingenone	3CLpro	*Triterygium regelii* Sprague & Takeda	Ryu et al. (2010)
Iguesterin	3CLpro	*Triterygium regelii* Sprague & Takeda	Ryu et al. (2010)
Sabadinine	3CLpro	*Veratrum sabadilla* Retz.	Toney et al. (2004)
Aurantiamide	Cathepsin-L	*Artemisia annua*	Wang et al. (2007)
Daidzein	GRP78 (HSPA5), estrogen receptor	*Cicer arietinum* L.	Sayed and Elfiky (2018)
Genistein	GRP78 (HSPA5), estrogen receptor	*Cicer arietinum* L.	Sayed and Elfiky (2018)
Formononetin	GRP78 (HSPA5), estrogen receptor	*Cicer arietinum* L.	Sayed and Elfiky (2018)
Biochanin A	GRP78 (HSPA5), estrogen receptor	*Cicer arietinum* L.	Sayed and Elfiky (2018)
Hydroxytyrosol	GRP78 (HSPA5)	Olive leaf	Sayed and Elfiky (2018)
*Cis*-*p*-coumaric acid	GRP78 (HSPA5)	*Gnetum cleistostachyum*	Sayed and Elfiky (2018)
Cinnamaldehyde	GRP78 (HSPA5)	*Cinnamomum*	Sayed and Elfiky (2018)
Thymoquinone	GRP78 (HSPA5)	*Nigella sativa*, *Monarda fistulosa*	Sayed and Elfiky (2018)
Griffithsin	Attachment, viral entry	Red algae *Griffithsia*	Dai et al. (2019), Moghaddam et al. (2014)
Agglutinin	Attachment, viral entry	*L. radiata*	Keyaerts et al. (2007)
GlcNAc-specific lectin	Attachment, viral entry	*Polyporus squamosus*	Keyaerts et al. (2007)
(GlcNAc)n-specific lectin	Attachment, viral entry	*Psathyrella velutina*	Keyaerts et al. (2007)
Gal-specific lectin	Attachment, viral entry	Mistletoe	Keyaerts et al. (2007)
Man/Glc-specific lectin	Attachment, viral entry	*Canavalia ensiformis*	Keyaerts et al. (2007)
Gal/GalNAc-specific lectin	Attachment, viral entry	*Erythrina corallodendron*	Keyaerts et al. (2007)
GalNAcα(1.3)Gal > GalNAc > Gal-specific lectin	Attachment, viral entry	*Artocarpus lakoocha*	Keyaerts et al. (2007)
Man/GalNAc-specific lectin	Attachment, viral entry	*Chlorophyllum molybdites *	Keyaerts et al. (2007)
Agglutinin	Replication, S-glycoprotein, GlcNAc	*Urtica dioica* L., *Galanthus nivalis* L	Kumaki et al. (2011)
Lectin	Propagation	*Allium porrum* L., *Nicotiana tabacum* L., *U. dioica*	Yonesi and Rezazadeh (2020)
Griffithsin	S-glycoprotein	*Griffithsia* sp.	O’Keefe et al. (2010), Millet et al. (2016), Li et al. (2019)
Concanavalin A	Hemagglutinate	*Canavalia ensiformis* (L.) DC.	Greig and Bouillant (1977)
DLasiL	Attachment, viral entry	*Dioclea lasiocarpa* Mart. ex Benth.	Hsieh et al. (2010)
Agglutinin	Hemagglutinate	*Galanthus nivalis* L.	Hsieh et al. (2010)
Lectin	Virion membrane	*Sambucus nigra* L.	Chen et al. (2014)
Aqueous extracts	3CLpro, RdRp	*Houttuynia cordata* Thunb.	Yang et al. (2010), Hoever et al. (2005)
Aqueous extracts	Replication	*Toona sinensis* (Juss.) M.Roem.	Jeong et al. (2014)
Methanol extracts	RdRp, protease	*Sophora* sp., *Acanthopanacis* sp., *Sanguisorbae* sp., *Torilis* sp.	Jin et al. (2019)
Ethanol extracts	Replication	*Nigella sativa* L*.*, *Citrus sinensis* L.	Jin et al. (2019)
Aqueous extracts	Attachment, viral entry	*Sambucus nigra* L. extract (black elderberry)	Wittemer et al. (2005)
Ethanolic extract	3CLpro	*Torreya nucifera*	Ryu et al. (2010)
Ethanol extracts	Viral attachment	*Pelargonium*	Michaelis et al. (2011)
Ethanol extracts	Virus envelope	*Mentha piperita* L., *Thymus vulgaris* L., *Desmodium canadense* (L.) DC.	Lelesius et al. (2019)

### Relationship Between Structures and Activities of Natural Products

The anti–SARS-CoV-2 natural compounds have been subjected to screening for understanding their structure–activity relationships (SARs). A possible approach to understand the SARs and inhibitory mechanism(s) is to resolve the inhibitor–target complex by using analytic tools. For example, crystallized complexes of the natural products and target proteins such as enzymes, surface proteins, and host receptors can be instrumentally analyzed. However, information on the successful SARs and the inhibitory mechanism(s) are currently limited. Instead, using molecular *in silico* ducking simulation and computational analysis, the SAR results have been reported. Using molecular modeling and docking techniques, potential binding abilities of the compounds to the pocket sites, interface sites, or catalytic sites of targets including proteases and the ACE2–S-glycoprotein complex have been suggested. The functional groups of the binding pocket interact with targets in van der Waals, hydrophilic, hydrophobic, and H-bond interactions.

As regards natural anthraquinones, rings and substituted glycosides differentially inhibit SARS-CoV-2 targets (Li and Jiang, 2018). For example, dihydroxyanthraquinone with C1 and C2−OH groups differently inhibit SARS-CoV-2 infection (Li and Jiang, 2018). Anthocyanins interact with the active site pockets of Mpro and human ACE2, where the active site of Mpro is polar in its chemical property, having affordable binding energies. Delphinidin, an anthocyanin derivative, forms H-bond in the binding site and π stacking with the hydrophobic pocket. A diglycosidic anthocyanin, delphinidin 3,5-diglucoside binds to the Mpro and ACE2 (Sharma and Shanavas, 2020), where it recognizes the flavylium nucleus ring and the Mpro catalytic site. In addition, the −OH groups of the phenyl ring recognize the Mpro S1 catalytic site through H-bonds (Sharma and Shanavas, 2020). The benzene ring and the Hie41 of the hydrophobic Mpro S2 domain form the p–π interaction. The −OH group of the flavylium nucleus binds to the Mpro S4, while the −OH groups of the benzoyl moiety of non-glycosidic 3,5-di-O-galloylshikimic acid form H-bonds with the Mpro cavity site. The OH- group and oxygen atoms of the benzoyl groups bind to the Mpro cavity site (Sharma and Shanavas, 2020). In contrast, for ACE2, oxygen of the COOH of 5-di-O-galloylshikimic acid binds to the Mpro cavity site via H-bonds, and non-covalent and ionic interactions. The −OH groups of the benzoyl moiety form H-bonds with the Mpro cavity site, where the side chain groups bind to benzoyl rings via the p–π stacking interaction. Therefore, the −OH groups are crucial for the SAR.

Flavones such as apigenin and quercetin inhibit 3CLpro activity, which coincides with the enzyme-inhibitory data. The 3CLpro inhibitory potential of biflavone with apigenin residue at the flavone C-3ʹ is enhanced, indicating that the 3CLpro inhibitory activity is upregulated by the additional apigenin residue at C-3′. In fact, the biflavonoid amentoflavone inhibits the 3CLpro activity. Quercetin recognizes the S-glycoprotein–ACE2 interface site (Smith and Smith, 2020; Williamson and Kerimi, 2020). A quercetin derivative, avicularin (Fukunaga et al., 1989) has also the Mpro-binding affinity. A similar scutellarein glucoside has the Mpro- and ACE2-binding affinities, where the −OH groups of glycoside form the H-bonds with the Mpro catalytic site. Another −OH group of the phenyl ring also forms H-bond with the Mpro. The phenyl ring also forms the π–π stacking interaction. The carbonyl oxygen and −OH group of the chromone nucleus form the H-bonds (Sharma and Shanavas, 2020). Similar to delphinidin diglucoside and scutellarein glucoside, l-arabinoside of avicularin binds to the catalytic site through H-bonds. The benzene ring involves in the π–π stacking with the hydrophobic subsite. Also, the −OH group of the chromone nucleus and benzene ring recognize the active site through H-bonds. The −OH groups of the arabinoside and phenyl ring recognize Mpro domain 1. Multiple *π*–π stacking interactions are formed between the chromone nucleus and the Mpro domain. The carbonyl group of the main nucleus forms the H-bonds with the Mpro. Likely, a flavanone glycoside, hesperidin, forms multiple H-bonds with the Mpro.

The flavonoid myricetin binds to both nsp13 and anti-3CLpro (Ananda Silva et al., 2020) as well as the TMPRSS2 active pocket through the 3 H-bonds, van der Waals forces, and π-anion (Pooja et al., 2021). Similarly, baicalein interacts with TMPRSS2 via 3 H-bonds, and van der Waals and *π*-stacking interactions. Aesculitannin B (Pooja et al., 2021) and proanthocyanidin bind to the TMPRSS2 active site via 5 H-bonds, and van der Waals and amide–π stacking interaction. Hydrocinnamic caffeic acid and ferulic acid recognize the Mpro active site via the H-bonds. Caffeic acid forms the H-bonds with both E- and N-proteins (Bhowmik et al., 2020). A bioflavonoid rutin also forms H-bonds with M- and N-proteins. Theaflavin interacts with the catalytic pocket groove near the RdRp active site through H-bonds and π–cation interaction, resulting in low docking score (Lung et al., 2020). Membrane binding of the alkyl gallates depends on alkyl chain lengths (Stefaniu et al., 2020) by high polarity–triggered reactivity. Therefore, the position of the −OH groups on the benzoic acid ring seems to be essential, compared with the number or type of ester, −OH, and methoxy groups.

For the SAR of glycyrrhizin and glycyrrhetinic acid, the free −OH (C-3), carbonyl (C-11), and COOH (C-30) groups influence the antiviral activity, while esterification of the −OH group on C-3 or the COOH group on C-30 decreases the activity. In addition, the dual esterification in the C-3 and C-30 decreases the activity, while substitution of the C-30 increases the activity (Wang et al., 2012). Betulonic acid, a triterpenoid, has an anti–SARS-CoV activity through the ketoxime backbone (Kazakova et al., 2011). The betulonic acid has a −OH and a COOH with a double bond at position C-20, 3-OH and 28-COOH groups (Regueiro-Ren et al., 2018), and C-3 and C-17 positions are crucial for the activity. Polyphenolic tannins show different binding capacities to the 3CLpro due to their SAR activities. The tannins recognize the receptor-binding spot and putative catalytic dyad of the 3CLpro. Tannic acid is a specific polyphenolic form of tannin with weak acidic properties due to the grouped phenols, where −OH groups, ketone groups (=O), and phenolic rings involve in binding to the 3CLpro through H-bonds and other forces (Khalifa et al., 2020). For example, hydrolyzable tannins including pedunculagin directly recognize the catalytic dyads and 3CLpro receptor–binding site with 5 H-bonds. Similarly, castalin and tercatain recognize the 3CLpro receptor–binding site via H-bonds and arene–arene interactions, influencing the catalytic dyad residues. Other hydrolyzable tannins including punicalin and isoterchebin secondarily recognize the catalytic dyad residues of the 3CLpro. Thymoquinone also interacts with the catalytic site of the 3CLpro via multiple H-bonds and π–H interactions (Kadil. et al., 2020). The −OH and carbonyl groups interact with the targets via H-bonds. For example, the −OH group binds to the Mpro, Nsp15, and S-glycoprotein (Kodchakorn et al., 2020).

## Conclusion

The COVID-19 outbreak is a global pandemic health problem. The SARS-CoV-2 RNA sequence has been known to be highly homologous with those of the CoVs. For the present crisis of pandemic SARS-CoV-2 infections, therapeutic and preventive approaches are simultaneously required to overcome the current life-threatening disease. Because development of blockers and inhibitors of viral entry and replication is urgent, computational AI has been incorporated to accelerate drug designation. Natural resources contain promising ligands for the development of therapeutic targets. Naturally occurring compounds are potentially promising resources for their antiviral properties. SARS-targeting agents can be effective against related CoV strains due to their similar life cycles. LMW compounds can be generated, discovered, and simulated with AI assistance for target molecules. Chemical derivative modification of known structures by AI-based technologies can enhance such drug activities. Thus, natural products may be useful for use in medical therapy of SARS-CoV-2 infections.

## Glossary

COVID-19: coronavirus disease 2019
SARS-CoV: severe acute respiratory syndrome-related coronavirus
MERS-V: Middle East respiratory syndrome virus
HCoV: human CoV
HE: hemagglutinin-esterase
RdRp: RNA-dependent RNA polymerase
E: envelope
N: nucleocapsid
S: spike glycoprotein
M: membrane matrix glycoprotein
ER: endoplasmic reticulum
ERGIC: endoplasmic reticulum–Golgi intermediate compartment
Nsp: nonstructural protein
TM: transmembrane
PLpro: Papain-like protease
RBD: receptor-binding domain
ssRNA: single-stranded RNA
NA: neuraminidase
Neu5Ac: N-acetylneuraminic acid
Neu5,9Ac2: 9-O-acetyl-N-acetylneuraminic acid
Neu5Ac9NAc: 9-acetamido-9-deoxy-N-acetylneuraminic acid
HEF: HE fusion protein
BCoV: Bovine CoV
MHV: murine hepatitis virus
CEACAM: carcinoembryonic antigen cell adhesion molecule
9-O-Ac-SA: 9-O-acetylated SA
IBV: infectious bronchitis virus
pAPN: porcine aminopeptidase N
GRP78: membrane-associated 78-kDa glucose-regulated protein
ACE2: angiotensin-converting enzyme 2
DPP4: dipeptidyl peptidase-4: dipeptidyl peptidase-4
hAPN: human APN
MHC-I: major histocompatibility complex class I
DC-SIGN: dendritic cell–specific intercellular adhesion molecule-3-grabbing nonintegrin
HSPG: heparan sulfate proteoglycan
HSPA5: heat shock protein A5
RAS: renin–angiotensin system
ADAM: A disintegrin and metallopeptidase domain
TACE: TNF-α–converting enzyme
TMPRSS: transmembrane protease serine
HAT: human airway trypsin-like protease
MSPL: serine 13
DPP4: dipeptidyl peptidase-4: dipeptidyl peptidase-4
BiP: immunoglobulin protein
UPR: unfolded protein response
ATF6: activating transcription factor 6
IRE1: inositol-requiring enzyme 1
PERK: protein kinase RNA (PKR)–like ER kinase
SBD: substrate-binding domain
PEDV: porcine epidemic diarrhea coronavirus virus
FIPV: feline infectious peritonitis virus
FeCV: feline enteric virus
GAG: glycosaminoglycan
RBM: receptor-binding motif
3CLpro: 3-chymotrypsin–like protease

## References

[B1] Adedeji A. O. Severson W. Jonsson C. Singh K. Weiss S. R. Sarafianos S. G. (2013). Novel Inhibitors of Severe Acute Respiratory Syndrome Coronavirus Entry that Act by Three Distinct Mechanisms. J. Virol. 87, 8017–8028. 10.1128/jvi.00998-13 23678171 PMC3700180

[B2] Adem Ş. Eyupoglu V. Sarfraz I. Rasul A. Zahoor A. F. Ali M. (2021). Caffeic Acid Derivatives (CAFDs) as Inhibitors of SARS-CoV-2: CAFDs-Based Functional Foods as a Potential Alternative Approach to Combat COVID-19. Phytomedicine. 85, 153310. 10.1016/j.phymed.2020.153310 32948420 PMC7442560

[B3] Alifano M. Alifano P. Forgez P. Iannelli A. (2020). Renin-angiotensin System at the Heart of COVID-19 Pandemic, Biochimie, 174, 30, 33. 10.1016/j.biochi.2020.04.008 32305506 PMC7161528

[B4] Anand K. Ziebuhr J. Wadhwani P. Mesters J. R. Hilgenfeld R. (2003). Coronavirus Main Proteinase (3CLpro) Structure: Basis for Design of Anti-SARS Drugs. Science 300(5626), 1763–1767. 10.1126/science.1085658 12746549

[B5] Ananda Silva A. A. Wiedemann L. S. M. Veiga-Junior V. F. (2020). Natural Products' Role against COVID-19. RSC Adv. 10(39), 23379–23393. 10.1039/D0RA03774E PMC9122563 35693131

[B6] Basu A. Sarkar A. Maulik U. (2020). Molecular Docking Study of Potential Phytochemicals and Their Effects on the Complex of SARS-CoV2 Spike Protein and Human ACE2. Sci. Rep. 10(1), 17699. 10.1038/s41598-020-74715-4 33077836 PMC7573581

[B7] Bhowmik D. Nandi R. Jagadeesan R. Kumar N. Prakash A. Kumar D. (2020).Identification of Potential Inhibitors against SARS-CoV-2 by Targeting Proteins Responsible for Envelope Formation and Virion Assembly Using Docking Based Virtual Screening, and Pharmacokinetics Approaches. Infect. Genet. Evol. 84, 104451. 10.1016/j.meegid.2020.104451 32640381 PMC7335633

[B8] Borah P. K. Chakraborty S. Jha A. N. Rajkhowa S. Duary R. K. (2016). In Silico approaches and Proportional Odds Model towards Identifying Selective ADAM17 Inhibitors from Anti-inflammatory Natural Molecules. J. Mol. Graphics Model. 70, 129–139. 10.1016/j.jmgm.2016.10.003 27723561

[B9] Cao J. Forrest J. C. Zhang X. (2015). A Screen of the NIH Clinical Collection Small Molecule Library Identifies Potential Anti-coronavirus Drugs. Antiviral Res *.* 114, 1–10. 10.1016/j.antiviral.2014.11.010 25451075 PMC7113785

[B11] Chan C.-M. Chu H. Wang Y. Wong B. H.-Y. Zhao X. Zhou J. (2016). Carcinoembryonic Antigen-Related Cell Adhesion Molecule 5 Is an Important Surface Attachment Factor that Facilitates Entry of Middle East Respiratory Syndrome Coronavirus. J. Virol. 90, 9114–9127. 10.1128/jvi.01133-16 27489282 PMC5044831

[B10] Chan C. M. Lau S. K. P. Woo P. C. Y. Tse H. Zheng B.-J. Chen L. (2009). Identification of Major Histocompatibility Complex Class I C Molecule as an Attachment Factor that Facilitates Coronavirus HKU1 Spike-Mediated Infection. Jvi 83, 1026–1035. 10.1128/jvi.01387-08 PMC2612401 18987136

[B12] Chan J. F.-W. Kok K.-H. Zhu Z. Chu H. To K. K.-W. Yuan S. (2020). Genomic Characterization of the 2019 Novel Human-Pathogenic Coronavirus Isolated from a Patient with Atypical Pneumonia after Visiting Wuhan. Emerging Microbes & Infections. 9(1), 221–236. 10.1080/22221751.2020.1719902 31987001 PMC7067204

[B13] Chandel V. Sharma P. P. Raj S. Choudhari R. Rathi B. Kumar D. (2020). Structure-based Drug Repurposing for Targeting Nsp9 Replicase and Spike Proteins of Severe Acute Respiratory Syndrome Coronavirus 2. J. Biomol. Struct. Dyn. 38, 1–14. 10.1080/07391102.2020.1811773 PMC7484568 32838660

[B16] Chen C.-J. Michaelis M. Hsu H.-K. Tsai C.-C. Yang K. D. Wu Y.-C. (2008). Toona Sinensis Roem Tender Leaf Extract Inhibits SARS Coronavirus Replication. J. Ethnopharmacology 120, 108–111. 10.1016/j.jep.2008.07.048 PMC7127248 18762235

[B15] Chen C.-N. Lin C. P. C. Huang K.-K. Chen W.-C. Hsieh H.-P. Liang P.-H. (2005). Inhibition of SARS-CoV 3C-like Protease Activity by Theaflavin-3,3'-Digallate (TF3). Evidence-Based Complement. Altern. Med. 2(2), 209–215. 10.1093/ecam/neh081 PMC1142193 15937562

[B17] Chen C. Zuckerman D. M. Brantley S. Sharpe M. Childress K. Hoiczyk E. (2014). *Sambucus Nigra* Extracts Inhibit Infectious Bronchitis Virus at an Early Point during Replication. BMC Vet. Res. 10, 24. 10.1186/1746-6148-10-24 24433341 PMC3899428

[B18] Chen H. Lin H. Xie S. Huang B. Qian Y. Chen K. 2019). Myricetin Inhibits NLRP3 Inflammasome Activation via Reduction of ROS-dependent Ubiquitination of ASC and Promotion of ROS-independent NLRP3 Ubiquitination. Toxicol. Appl. Pharmacol. 365, 19–29. 10.1016/j.taap.2018.12.019 30594691

[B19] Chen I.-Y. Moriyama M. Chang M.-F. Ichinohe T. (2019). Severe Acute Respiratory Syndrome Coronavirus Viroporin 3a Activates the NLRP3 Inflammasome. Front. Microbiol. 10(50), 10.3389/fmicb.2019.00050 PMC6361828 30761102

[B14] Chen M. Theander T. G. Christensen S. B. Hviid L. Zhai L. Kharazmi A. (1994). Licochalcone A, a New Antimalarial Agent, Inhibits In Vitro Growth of the Human Malaria Parasite Plasmodium Falciparum and Protects Mice from P. Yoelii Infection. Antimicrob. Agents Chemother. 38 (7), 1470–1475. 10.1128/aac.38.7.1470 7979274 PMC284578

[B21] Cheng L. Zheng W. Li M. Huang J. Bao S. Xu Q. (2020). Citrus Fruits Are Rich in Flavonoids for Immunoregulation and Potential Targeting ACE2. Preprints 2020020313 10.1007/s13659-022-00325-4 PMC8844329 35157175

[B20] Cheng P.-W. Ng L.-T. Chiang L.-C. Lin C.-C. (2006). Antiviral Effects of Saikosaponins on Human Coronavirus 229E In Vitro. Clin. Exp. Pharmacol. Physiol. 33(7), 612–616. 10.1111/j.1440-1681.2006.04415.x 16789928 PMC7162031

[B22] Chiang L. C. Ng L. T. Liu L. T. Shieh D. E. Lin C. C. (2003). Cytotoxicity and Anti-hepatitis B Virus Activities of Saikosaponins from Bupleurum Species. Planta Med. 69(08), 705–709. 10.1055/s-2003-42797 14531019

[B23] Chiow K. H. Phoon M. C. Putti T. Tan B. K. H. Chow V. T. (2016). Evaluation of Antiviral Activities of Houttuynia Cordata Thunb. Extract, Quercetin, Quercetrin and Cinanserin on Murine Coronavirus and Dengue Virus Infection. Asian Pac. J. Trop. Med. 9(1), 1–7. 10.1016/j.apjtm.2015.12.002 26851778 PMC7104935

[B24] Cho J. H. Bernard D. L. Sidwell R. W. Kern E. R. Chu C. K. (2006). Synthesis of Cyclopentenyl Carbocyclic Nucleosides as Potential Antiviral Agents against Orthopoxviruses and SARS. J. Med. Chem. 49(3), 1140–1148. 10.1021/jm0509750 16451078

[B25] Cho J. K. Curtis-Long M. J. Lee K. H. Kim D. W. Ryu H. W. Yuk H. J. (2013). Geranylated Flavonoids Displaying SARS-CoV Papain-like Protease Inhibition from the Fruits of *Paulownia Tomentosa* . Bioorg. Med. Chem. 21(11), 3051–3057. 10.1016/j.bmc.2013.03.027 23623680 PMC7126831

[B26] Choe J.-Y. Kim S.-K. (2017). Quercetin and Ascorbic Acid Suppress Fructose-Induced NLRP3 Inflammasome Activation by Blocking Intracellular Shuttling of TXNIP in Human Macrophage Cell Lines. Inflammation 40(3), 980–994. 10.1007/s10753-017-0542-4 28326454

[B27] Choi H.-J. Kim J.-H. Lee C.-H. Ahn Y.-J. Song J.-H. Baek S.-H. (2009). Antiviral Activity of Quercetin 7-rhamnoside against Porcine Epidemic Diarrhea Virus. Antiviral Res. 81(1), 77–81. 10.1016/j.antiviral.2008.10.002 18992773 PMC7114206

[B28] Cinatl J. Morgenstern B. Bauer G. Chandra P. Rabenau H. Doerr H. (2003). Glycyrrhizin, an Active Component of Liquorice Roots, and Replication of SARS-Associated Coronavirus. The Lancet 361, 2045–2046. 10.1016/s0140-6736(03)13615-x PMC7112442 12814717

[B29] Covés-Datson E. M. Dyall J. DeWald L. E. King S. R. Dube D. Legendre M. (2019). Inhibition of Ebola Virus by a Molecularly Engineered Banana Lectin. Plos Negl. Trop. Dis. 13(7), e0007595. 10.1371/journal.pntd.0007595 31356611 PMC6687191

[B30] Dai W. Bi J. Li F. Wang S. Huang X. Meng X. 2019). Antiviral Efficacy of Flavonoids against Enterovirus 71 Infection In Vitro and in Newborn Mice. Viruses 11(7), 625. 10.3390/v11070625 PMC6669683 31284698

[B31] Dao T. T. Nguyen P. H. Lee H. S. Kim E. Park J. Lim S. I. (2011). "Chalcones as Novel Influenza A (H1N1) Neuraminidase Inhibitors from *Glycyrrhiza Inflata* ". Bioorg. Med. Chem. Lett. 21 (1), 294–298. 10.1016/j.bmcl.2010.11.016 21123068

[B32] de Vries A. A. F. Horzinek M. C. Rottier P. J. M. de Groot R. J. (1997). The Genome Organization of the Nidovirales: Similarities and Differences between Arteri-, Toro-, and Coronaviruses. Semin. Virol. 8, 33–47. 10.1006/smvy.1997.0104 32288441 PMC7128191

[B33] Delmas B. Gelfi J. L'Haridon R. Vogel L. K. Sjöström H. Norén O. (1992). Aminopeptidase N Is a Major Receptor for the Enteropathogenic Coronavirus TGEV. Nature 357(6377), 417–420. 10.1038/357417a0 1350661 PMC7095137

[B34] Deng F. Ye G. Liu Q. Navid M. Zhong X. Li Y. (2016). Identification and Comparison of Receptor Binding Characteristics of the Spike Protein of Two Porcine Epidemic Diarrhea Virus Strains. Viruses 8(3):55. 10.3390/v8030055 26907329 PMC4810246

[B35] Earnest J. T. Hantak M. P. Li K. McCray P. B. Jr. Perlman S. Gallagher T. (2017). The Tetraspanin CD9 Facilitates MERS-Coronavirus Entry by Scaffolding Host Cell Receptors and Proteases. Plos Pathog. 13(7), e1006546. 10.1371/journal.ppat.1006546 28759649 PMC5552337

[B36] Elfiky A. A. (2020). Ebola Virus Glycoprotein GP1-Host Cell-Surface HSPA5 Binding Site Prediction. Cell Stress and Chaperones. 25(3), 541–548. 10.1007/s12192-020-01106-z 32291698 PMC7154572

[B37] Elfiky A. A. (2020). Natural Products May Interfere with SARS-CoV-2 Attachment to the Host Cell. J. Biomol. Struct. Dyn. 1, 1–10. 10.1080/07391102.2020.1761881 PMC7212544 32340551

[B38] Erlina L. Paramita R. I. Kusuma W. A. Fadilah F. Tedjo A. Pratomo I. P. (2020). Virtual Screening on Indonesian Herbal Compounds as COVID-19 Supportive Therapy: Machine Learning and Pharmacophore Modeling Approaches. BMC Med. Inform. Decis. Making. 10.21203/rs.3.rs-29119/v1 PMC9347098 35922786

[B39] Fitriani I. N. Utami W. Zikri A. T. Santoso P. (2020), In Silico Approach of Potential Phytochemical Inhibitor from Moringa Oleifera, Cocos Nucifera, Allium cepa, Psidium Guajava, and *Eucalyptus* Globulus for the Treatment of COVID-19 by Molecular Docking. 10.21203/rs.3.rs-42747/v1

[B40] Fu S. Xu L. Li S. Qiu Y. Liu Y. Wu Z. (2016). Baicalin Suppresses NLRP3 Inflammasome and Nuclear Factor-Kappa B (NF-Κb) Signaling during *Haemophilus Parasuis* Infection. Vet. Res. 47(1), 80. 10.1186/s13567-016-0359-4 27502767 PMC4977663

[B41] Fu X. Wang Z. Li L. Dong S. Li Z. Jiang Z. (2016). Novel Chemical Ligands to Ebola Virus and Marburg Virus Nucleoproteins Identified by Combining Affinity Mass Spectrometry and Metabolomics Approaches. Sci. Rep. 6, 29680. 10.1038/srep29680 27403722 PMC4940736

[B42] Fukunaga T. Nishiya K. Kajikawa I. Takeya K. Itokawa H. (1989). Studies on the Constituents of Japanese Mistletoes from Different Host Trees, and Their Antimicrobial and Hypotensive Properties. Chem. Pharm. Bull. Bulletin *,* 37, 1543–1546. 10.1248/cpb.37.1543 2776238

[B43] Gheblawi M. Wang K. Viveiros A. Nguyen Q. Zhong J.-C. Turner A. J. (2020). Angiotensin-Converting Enzyme 2: SARS-CoV-2 Receptor and Regulator of the Renin-Angiotensin System. Circ. Res. 126(10), 1456–1474. 10.1161/circresaha.120.317015 32264791 PMC7188049

[B44] Ghosh A. K. Takayama J. Rao K. V. Ratia K. Chaudhuri R. Mulhearn D. C. (2010). Severe Acute Respiratory Syndrome Coronavirus Papain-like Novel Protease Inhibitors: Design, Synthesis, Protein−Ligand X-Ray Structure and Biological Evaluation. J. Med. Chem., 53, 4968–4979. 10.1021/jm1004489 20527968 PMC2918394

[B45] Greig A. S. Bouillant A. M. (1977). Binding Effects of Concanavalin A on a Coronavirus. Can. J. Comp. Med. 41(1), 122–126. 832184 PMC1277703

[B46] Haga S. Nagata N. Okamura T. Yamamoto N. Sata T. Yamamoto N. (2010). TACE Antagonists Blocking ACE2 Shedding Caused by the Spike Protein of SARS-CoV Are Candidate Antiviral Compounds. Antiviral Res. 85, 551–555. 10.1016/j.antiviral.2009.12.001 19995578 PMC7114272

[B47] Haiying L. Na H. Xiaoyuan X. (2003). The Curative Effects of Glycyrrhizin on Patients with SARS. Annual Meeting of The Society of Infectious and Parasitic Diseases, Chinese Medical Association, Wuhan, China, pp. 18–22.

[B48] Häkkinen S. H. Kärenlampi S. O. Heinonen I. M. Mykkänen H. M. Törrönen A. R. (1999). Content of the Flavonols Quercetin, Myricetin, and Kaempferol in 25 Edible Berries. J. Agric. Food Chem. 47(6), 2274–2279. 10.1021/jf9811065 10794622

[B49] Han D. P. Lohani M. Cho M. W. (2007). Specific Asparagine-Linked Glycosylation Sites Are Critical for DC-SIGN- and L-SIGN-Mediated Severe Acute Respiratory Syndrome Coronavirus Entry. J. Virol. 81(21), 12029–12039. 10.1128/jvi.00315-07 17715238 PMC2168787

[B50] Heino S. Lusa S. Somerharju P. Ehnholm C. Olkkonen V. M. Ikonen E. (2000). Dissecting the Role of the Golgi Complex and Lipid Rafts in Biosynthetic Transport of Cholesterol to the Cell Surface. Proc. Natl. Acad. Sci. 97, 8375–8380. 10.1073/pnas.140218797 10890900 PMC26955

[B51] Heurich A. Hofmann-Winkler H. Gierer S. Liepold T. Jahn O. Pohlmann S. (2014). TMPRSS2 and ADAM17 Cleave ACE2 Differentially and Only Proteolysis by TMPRSS2 Augments Entry Driven by the Severe Acute Respiratory Syndrome Coronavirus Spike Protein. J. Virol. 88(2), 1293–1307. 10.1128/jvi.02202-13 24227843 PMC3911672

[B52] Ho T. Wu S. Chen J. Li C. Hsiang C. (2007). Emodin Blocks the SARS Coronavirus Spike Protein and Angiotensin-Converting Enzyme 2 Interaction. Antiviral Res. 74(2), 92–101. 10.1016/j.antiviral.2006.04.014 16730806 PMC7114332

[B53] Hoever G. Baltina L. Michaelis M. Kondratenko R. Baltina L. Tolstikov G. A. (2005). Antiviral Activity of Glycyrrhizic Acid Derivatives against SARS−Coronavirus. J. Med. Chem. 48, 1256–1259. 10.1021/jm0493008 15715493

[B54] Hsieh L.-E. Lin C.-N. Su B.-L. Jan T.-R. Chen C.-M. Wang C.-H. (2010). Synergistic Antiviral Effect of *Galanthus Nivalis* Agglutinin and Nelfinavir against Feline Coronavirus. Antiviral Res. 88(1), 25–30. 10.1016/j.antiviral.2010.06.010 20603153 PMC7114315

[B55] Hulswit R. J. G. Lang Y. Bakkers M. J. G. Li W. Li Z. Schouten A. (2019). Human Coronaviruses OC43 and HKU1 Bind to 9-O-Acetylated Sialic Acids via a Conserved Receptor-Binding Site in Spike Protein Domain A. Proc. Natl. Acad. Sci. USA. 116:2681–2690. 10.1073/pnas.1809667116 30679277 PMC6377473

[B56] Hwang H.-J. Han J.-W. Jeon H. Cho K. Kim J.-h. Lee D.-S. (2020). Characterization of a Novel Mannose-Binding Lectin with Antiviral Activities from Red Alga, *Grateloupia Chiangii* . Biomolecules 10(2), 333. 10.3390/biom10020333 PMC7072537 32092955

[B57] Ibrahim I. M. Abdelmalek D. H. Elfiky A. A. (2019). GRP78: a Cell's Response to Stress. Life Sci *.* 226, 156–163. 10.1016/j.lfs.2019.04.022 30978349 PMC7094232

[B58] Ibrahim I. M. Abdelmalek D. H. Elshahat M. E. Elfiky A. A. (2020). COVID-19 Spike-Host Cell Receptor GRP78 Binding Site Prediction. J. Infect. 80(5), 554–562. 10.1016/j.jinf.2020.02.026 32169481 PMC7102553

[B59] Im K. Kim J. Min H. (2015). Ginseng, the Natural Effectual Antiviral: Protective Effects of Koran Red Ginseng against Viral Infection 40(4):309–314. 10.1016/j.jgr.2015.09.002 Available at: https://www.ncbi.nlm.nih.gov/pmc/articles/PMC5052424/ PMC5052424 27746682

[B60] Islam M. T. Sarkar C. El‐Kersh D. M. Jamaddar S. Uddin S. J. Shilpi J. A. (2020). Natural Products and Their Derivatives against Coronavirus: A Review of the Non‐clinical and Pre‐clinical Data. Phytotherapy Res. 34(10), 2471–2492. 10.1002/ptr.6700 32248575

[B61] Jeong H.-U. Kwon S.-S. Kong T. Y. Kim J. H. Lee H. S. (2014). "Inhibitory Effects of Cedrol, β-Cedrene, and Thujopsene on Cytochrome P450 Enzyme Activities in Human Liver Microsomes". J. Toxicol. Environ. Health A 77 (22–24): 1522–1532. 10.1080/15287394.2014.955906 25343299

[B62] Jin Y. H. Kwon S. Choi J. G. Cho W. K. Lee B. Ma J. Y. (2019). Toosendanin from *Melia Fructus* Suppresses Influenza A Virus Infection by Altering Nuclear Localization of Viral Polymerase PA Protein. Front. Pharmacol. 10, 1025. 10.3389/fphar.2019.01025 31607903 PMC6757512

[B63] Jin Z. Du X. Xu Y. Deng Y. Liu M. Zhao Y. 2020). Structure of Mpro from SARS-CoV-2 and Discovery of its Inhibitors. Nature, 582, 289, 293., 10.1038/s41586-020-2223-y 32272481

[B64] Joshi R. S. Jagdale S. S. Bansode S. B. Shankar S. S. Tellis M. B. Pandya V. K. (2020). Discovery of Potential Multi-Target-Directed Ligands by Targeting Host-specific SARS-CoV-2 Structurally Conserved Main Protease. J. Biomol. Struct. Dyn. 2020, 1–16. 10.1080/07391102.2020.1760137 PMC7212545 32329408

[B65] Kadil Y. Mouhcine M. Filali H. (2020). In Silico Investigation of the SARS CoV2 Protease with Thymoquinone Major Constituent of Nigella Sativa. Cddt, 17, 12. 10.2174/1570163817666200712164406 32652915

[B66] Kang K. B. Ming G. Kim G. J. Ha T.-K. -Q. Choi H. Oh W. K. (2015). Jubanines F-J, Cyclopeptide Alkaloids from the Roots of *Ziziphus Jujuba* . Phytochemistry 119, 90–95. 10.1016/j.phytochem.2015.09.001 26361730 PMC7111685

[B500] Kalhori M. R. Saadatpour F. Arefian E. Soleiman M. Farzaei M. H. Aneva I. Y. (2021). The Potential Therapeutic Effect of RNA Interference and Natural Products on COVID-19: A Review of the Coronaviruses Infection. Front. Pharmacol. 12, 616993. 10.3389/fphar.2021.616993 33716745 PMC7953353

[B67] Kazakova O. B. Giniiatullina G. V. Tolstikov G. A. (2011). Synthesis of A-Secomethylenamino- and Substituted Amidoximotriterpenoids. Bioorg. Khim. 37(5), 690–696. 10.1134/s1068162011050086 22332366 PMC7088524

[B68] Keyaerts E. Vijgen L. Pannecouque C. Van Damme E. Peumans W. Egberink H. (2007). Plant Lectins Are Potent Inhibitors of Coronaviruses by Interfering with Two Targets in the Viral Replication Cycle. Antiviral Res. 75(3), 179–187. 10.1016/j.antiviral.2007.03.003 17428553 PMC7114093

[B69] Khaerunnisa S. Kurniawan H. Awaluddin R. Suhartati S. Soetjipto S. (2020). Potential Inhibitor of COVID-19 Main Protease (Mpro) from Several Medicinal Plant Compounds by Molecular Docking Study. Preprints 2020, 2020030226. 10.20944/preprints202003.0226.v1

[B70] Khalifa I. Zhu W. Mohammed H. H. H. Dutta K. Li C. (2020). Tannins Inhibit SARS‐CoV‐2 through Binding with Catalytic Dyad Residues of 3CL Pro : An In Silico Approach with 19 Structural Different Hydrolysable Tannins. J. Food Biochem., 44 e13432. 10.1111/jfbc.13432 PMC7435556 32783247

[B75] Kim C.-H. (2020). SARS-CoV-2 Evolutionary Adaptation toward Host Entry and Recognition of Receptor O-Acetyl Sialylation in Virus-Host Interaction. Ijms 21(12), 4549. 10.3390/ijms21124549 PMC7352545 32604730

[B74] Kim D. Min J. Jang M. Lee J. Shin Y. Park C. (2019). Natural Bis-Benzylisoquinoline Alkaloids-Tetrandrine, Fangchinoline, and Cepharanthine, Inhibit Human Coronavirus OC43 Infection of MRC-5 Human Lung Cells. Biomolecules 9(11), 696. 10.3390/biom9110696 PMC6921063 31690059

[B73] Kim D. W. Seo K. H. Curtis-Long M. J. Oh K. Y. Oh J.-W. Cho J. K. (2014). Phenolic Phytochemical Displaying SARS-CoV Papain-like Protease Inhibition from the Seeds of *Psoralea Corylifolia* . J. Enzyme Inhib. Med. Chem. 29(1), 59–63. 10.3109/14756366.2012.753591 23323951

[B72] Kim H.-Y. Eo E.-Y. Park H. Kim Y.-C. Park S. Shin H.-J. (2010). Medicinal Herbal Extracts of Sophorae Radix, Acanthopanacis Cortex, Sanguisorbae Radix and Torilis Fructus Inhibit Coronavirus Replication In Vitro. Antivir. Ther. 15(5), 697–709. 10.3851/imp1615 20710051

[B71] Kim H.-Y. Shin H.-S. Park H. Kim Y.-C. Yun Y. G. Park S. (2008). *In vitro* inhibition of Coronavirus Replications by the Traditionally Used Medicinal Herbal Extracts, Cimicifuga Rhizoma, Meliae Cortex, Coptidis Rhizoma, and Phellodendron Cortex. J. Clin. Virol. 41(2), 122–128. 10.1016/j.jcv.2007.10.011 18036887 PMC7108295

[B76] Kodchakorn K. Poovorawan Y. Suwannakarn K. Kongtawelert P. (2020). Molecular Modelling Investigation for Drugs and Nutraceuticals against Protease of SARS-CoV-2. J. Mol. Graphics Model. 101, 107717. 10.1016/j.jmgm.2020.107717 PMC7434411 32861974

[B77] Koehn F. E. Sarath G. P. Neil D. N. Cross S. S. (1991). Halitunal, an Unusual Diterpene Aldehyde from the Marine Alga *Halimeda Tuna* . Tetrahedron Lett. 32(2), 169–172. 10.1016/0040-4039(91)80845-w 32287435 PMC7125756

[B78] Kolodziej H. (2011). Antimicrobial, Antiviral and Immunomodulatory Activity Studies of *Pelargonium* Sidoides (EPs 7630) in the Context of Health Promotion. Pharmaceuticals, 4(10), 1295–1314. 10.3390/ph4101295 27721327 PMC4060126

[B79] Kumaki Y. Wandersee M. K. Smith A. J. Zhou Y. Simmons G. Nelson N. M. (2011). Inhibition of Severe Acute Respiratory Syndrome Coronavirus Replication in a Lethal SARS-CoV BALB/c Mouse Model by Stinging Nettle Lectin, Urtica Dioica Agglutinin. Antiviral Res. 90(1), 22–32. 10.1016/j.antiviral.2011.02.003 21338626 PMC3085190

[B80] Kumar V. Dhanjal J. K. Kaul S. C. Wadhwa R. Sundar D. (2020). Withanone and Caffeic Acid Phenethyl Ester Are Predicted to Interact with Main Protease (Mpro) of SARS-CoV-2 and Inhibit its Activity. J. Biomol. Struct. Dyn. 39, 1–13. 10.1080/07391102.2020.1772108 PMC7284143 32431217

[B81] Kwon H.-J. Ryu Y. B. Kim Y.-M. Song N. Kim C. Y. Rho M.-C. (2013). *In vitro* antiviral Activity of Phlorotannins Isolated from *Ecklonia Cava* against Porcine Epidemic Diarrhea Coronavirus Infection and Hemagglutination. Bioorg. Med. Chem. 21(15), 4706–4713. 10.1016/j.bmc.2013.04.085 23746631 PMC7127107

[B82] Langereis M. A. Zeng Q. Heesters B. A. Huizinga E. G. de Groot R. J. (2012). The Murine Coronavirus Hemagglutinin-Esterase Receptor-Binding Site: a Major Shift in Ligand Specificity through Modest Changes in Architecture. Plos Pathog. 8, e1002492. 10.1371/journal.ppat.1002492 22291594 PMC3266934

[B83] Lau K.-M. Lee K.-M. Koon C.-M. Cheung C. S.-F. Lau C.-P. Ho H.-M. (2008). Immunomodulatory and Anti-SARS Activities of *Houttuynia Cordata* . J. Ethnopharmacology 118, 79–85. 10.1016/j.jep.2008.03.018 PMC7126383 18479853

[B84] Lee J.-H. Park J.-S. Lee S.-W. Hwang S.-Y. Young B.-E. Choi H.-J. (2015). Porcine Epidemic Diarrhea Virus Infection: Inhibition by Polysaccharide from *Ginkgo Biloba* Exocarp and Mode of its Action. Virus. Res. 195, 148–152. 10.1016/j.virusres.2014.09.013 25300802

[B85] Lelesius R. Karpovaite A. Mickiene R. Drevinskas T. Tiso N. Ragazinskiene O. (2019). *In vitro* antiviral Activity of Fifteen Plant Extracts against Avian Infectious Bronchitis Virus. BMC Vet. Res. 15(1), 178. 31142304 10.1186/s12917-019-1925-6 PMC6540435

[B86] Letko M. Marzi A. Munster V. (2020). Functional Assessment of Cell Entry and Receptor Usage for SARS-CoV-2 and Other Lineage B Betacoronaviruses. Nat. Microbiol. 5(4), 562–569. 10.1038/s41564-020-0688-y 32094589 PMC7095430

[B91] Li F. (2015). Receptor Recognition Mechanisms of Coronaviruses: a Decade of Structural Studies. J. Virol. 89, 1954–1964. 10.1128/JVI.02615-14 25428871 PMC4338876

[B92] Li F. (2016). Structure, Function, and Evolution of Coronavirus Spike Proteins. Annu. Rev. Virol. 3, 237–261. 10.1146/annurev-virology-110615-042301 27578435 PMC5457962

[B89] Li H. Wu J. Zhang Z. Ma Y. Liao F. Zhang Y. (2011). Forsythoside a Inhibits the Avian Infectious Bronchitis Virus in Cell Culture. Phytother. Res. 25(3), 338–342. 10.1002/ptr.3260 20677175 PMC7168103

[B90] Li L. Yu X. Zhang H. Cheng H. Hou L. Zheng Q. (2019). *In vitro* antiviral Activity of Griffithsin against Porcine Epidemic Diarrhea Virus. Virus Genes 55(2), 174–181. 10.1007/s11262-019-01633-7 30637608 PMC7089098

[B88] Li S. Chen C. Zhang H. Guo H. Wang H. Wang L. (2005). Identification of Natural Compounds with Antiviral Activities against SARS-Associated Coronavirus. Antiviral Res. 67(1), 18–23. 10.1016/j.antiviral.2005.02.007 15885816 PMC7114104

[B87] Li Y. Jiang J.-G. (2018). Health Functions and Structure-Activity Relationships of Natural Anthraquinones from Plants. Food Funct. 9(12), 6063–6080. 10.1039/c8fo01569d 30484455

[B93] Liang Y. Zhang Q. Zhang L. Wang R. Xu X. Hu X. (2019). *Astragalus* Membranaceus Treatment Protects Raw264.7 Cells from Influenza Virus by Regulating G1 Phase and the TLR3-Mediated Signaling Pathway. Evid. Based Complement. Alternat. Med. 2019, 2971604. 10.1155/2019/2971604 31975996 PMC6955127

[B94] Lim H. Min D. S. Park H. Kim H. P. (2018). Flavonoids Interfere with NLRP3 Inflammasome Activation. Toxicol. Appl. Pharmacol. 355, 93–102. 10.1016/j.taap.2018.06.022 29960001

[B95] Lin C.-W. Tsai F.-J. Tsai C.-H. Lai C.-C. Wan L. Ho T.-Y. (2005). Anti-SARS Coronavirus 3C-like Protease Effects of Isatis Indigotica Root and Plant-Derived Phenolic Compounds. Antiviral Res. 68, 36–42. 10.1016/j.antiviral.2005.07.002 16115693 PMC7114321

[B96] Lin S. C. Ho C. T. Chuo W. H. Li S. Wang T. T. Lin C. C. (2017). Effective Inhibition of MERS-CoV Infection by Resveratrol. BMC Infect. Dis. 17(1), 144. 10.1186/s12879-017-2253-8 28193191 PMC5307780

[B97] Loizzo M. R. Saab A. M. Tundis R. Statti G. A. Menichini F. Lampronti I. (2008). Phytochemical Analysis Andin Vitro Antiviral Activities of the Essential Oils of Seven Lebanon Species. C&B. 5(3), 461–470. 10.1002/cbdv.200890045 PMC7161995 18357554

[B98] Lung J. Lin Y. S. Yang Y. H. Chou Y. L. Shu L. H. Cheng Y. C. (2020). The Potential Chemical Structure of anti‐SARS‐CoV‐2 RNA‐dependent RNA Polymerase. J. Med. Virol. 92(6), 693–697. 10.1002/jmv.25761 32167173 PMC7228302

[B99] Luo W. Su X. Gong S. Qin Y. Liu W. Li J. (2009). Anti-SARS Coronavirus 3C-like Protease Effects of Rheum Palmatum L. Extracts. Biosci. Trends 3(4), 124–126. 20103835

[B100] Mani J. S. Johnson J. B. Steel J. C. Broszczak D. A. Neilsen P. M. Walsh K. B. (2020). Natural Product-Derived Phytochemicals as Potential Agents against Coronaviruses: a Review. Virus. Res. 284, 197989. 10.1016/j.virusres.2020.197989 32360300 PMC7190535

[B300] Majnooni M. B. Fakhri S. Shokoohinia Y. Kiyani N. Stage K. Mohammadi P. (2020). Phytochemicals: Potential Therapeutic Interventions Against Coronavirus-Associated Lung Injury. Front. Pharmacol. 11, 1744. 10.3389/fphar.2020.588467 PMC7919380 33658931

[B101] Marzi A. Gramberg T. Simmons G. Moller P. Rennekamp A. J. Krumbiegel M. (2004). DC-SIGN and DC-SIGNR Interact with the Glycoprotein of Marburg Virus and the S Protein of Severe Acute Respiratory Syndrome Coronavirus. Jvi 78, 12090–12095. 10.1128/jvi.78.21.12090-12095.2004 PMC523257 15479853

[B102] Matsuyama S. Taguchi F. (2002). Receptor-induced Conformational Changes of Murine Coronavirus Spike Protein. J. Virol. 76, 11819–11826. 10.1128/jvi.76.23.11819-11826.2002 12414924 PMC136913

[B103] McCutcheon A. R. Roberts T. E. Gibbons E. Ellis S. M. Babiuk L. A. Hancock R. E. W. (1995). Antiviral Screening of British Columbian Medicinal Plants. J. Ethnopharmacology 49(2), 101–110. 10.1016/0378-8741(95)90037-3 PMC7131204 8847882

[B104] McDonagh P. Sheehy P. A. Norris J. M. (2014). Identification and Characterisation of Small Molecule Inhibitors of Feline Coronavirus Replication. Vet. Microbiol. 174(3-4), 438–447. 10.1016/j.vetmic.2014.10.030 25465182 PMC7117153

[B105] McKee D. L. Sternberg A. Stange U. Laufer S. Naujokat C. (2020). Candidate Drugs against SARS-CoV-2 and COVID-19. Pharmacol. Res., 157, 104859. 104859. 10.1016/j.phrs.2020.104859 32360480 PMC7189851

[B106] Meneguzzo F. Ciriminna R. Zabini F. Pagliaro M. (2020). Review of Evidence Available on Hesperidin-Rich Products as Potential Tools against COVID-19 and Hydrodynamic Cavitation-Based Extraction as a Method of Increasing Their Production. Processes 8, 549. 10.3390/pr8050549

[B107] Michaelis M. Doerr H. W. Cinatl J. Jr. (2011). Investigation of the Influence of EPs 7630, a Herbal Drug Preparation from *Pelargonium* Sidoides, on Replication of a Broad Panel of Respiratory Viruses. Phytomedicine 18(5), 384–386. 10.1016/j.phymed.2010.09.008 21036571 PMC7127141

[B108] Michaelis M. Geiler J. Naczk P. Sithisarn P. Leutz A. Doerr H. W. (2011). Glycyrrhizin Exerts Antioxidative Effects in H5N1 Influenza A Virus-Infected Cells and Inhibits Virus Replication and Pro-inflammatory Gene Expression. PLoS One 6, e19705. 10.1371/journal.pone.0019705 21611183 PMC3096629

[B109] Michelow I. C. Lear C. Scully C. Prugar L. I. Longley C. B. Yantosca L. M. (2011). High-dose Mannose-Binding Lectin Therapy for Ebola Virus Infection. J. Infect. Dis. 203(2), 175–179. 10.1093/infdis/jiq025 21288816 PMC3071052

[B110] Miean K. H. Mohamed S. (2001). Flavonoid (Myricetin, Quercetin, Kaempferol, Luteolin, and Apigenin) Content of Edible Tropical Plants. J. Agric. Food Chem. 49(6), 3106–3112. 10.1021/jf000892m 11410016

[B111] Mikulic-Petkovsek M. Slatnar A. Stampar F. Veberic R. (2012). HPLC-MSn Identification and Quantification of Flavonol Glycosides in 28 Wild and Cultivated Berry Species. Food Chem. 135(4), 2138–2146. 10.1016/j.foodchem.2012.06.115 22980782

[B112] Milewska A. Zarebski M. Nowak P. Stozek K. Potempa J. Pyrc K. (2014). Human Coronavirus NL63 Utilizes Heparan Sulfate Proteoglycans for Attachment to Target Cells. J. Virol. 88, 13221–13230. 10.1128/jvi.02078-14 25187545 PMC4249106

[B113] Millet J. K. Séron K. Labitt R. N. Danneels A. Palmer K. E. Whittaker G. R. (2016). Middle East Respiratory Syndrome Coronavirus Infection Is Inhibited by Griffithsin. Antiviral Res. 133, 1–8. 10.1016/j.antiviral.2016.07.011 27424494 PMC7113895

[B114] Moghaddam E. Teoh B. T. Sam S. S. Lani R. Hassandarvish P. Chik Z. (2014). Baicalin, a Metabolite of Baicalein with Antiviral Activity against Dengue Virus. Sci. Rep. 4, 5452. 10.1038/srep05452 24965553 PMC4071309

[B116] Müller C. Obermann W. Schulte F. W. Lange-Grünweller K. Oestereich L. Elgner F. (2020). Comparison of Broad-Spectrum Antiviral Activities of the Synthetic Rocaglate CR-31-B (−) and the eIF4A-Inhibitor Silvestrol. Antiviral Res. 175, 104706. 10.1016/j.antiviral.2020.104706 31931103 PMC7114339

[B115] Müller C. Schulte F. W. Lange-Grünweller K. Obermann W. Madhugiri R. Pleschka S. (2018). Broad-spectrum Antiviral Activity of the eIF4A Inhibitor Silvestrol against Corona- and Picornaviruses. Antiviral Res. 150, 123–129. 10.1016/j.antiviral.2017.12.010 29258862 PMC7113723

[B117] Naskalska A. Dabrowska A. Szczepanski A. Milewska A. Jasik K. P. Pyrc K. (2019). Membrane Protein of Human Coronavirus NL63 Is Responsible for Interaction with the Adhesion Receptor. J. Virol. 93(19), e00355-19. 10.1128/JVI.00355-19 31315999 PMC6744225

[B118] Nguyen P. Q. T. Ooi J. S. G. Nguyen N. T. K. Wang S. Huang M. Liu D. X. (2015). Antiviral Cystine Knot α-Amylase Inhibitors from *Alstonia scholaris* . J. Biol. Chem. 290(52), 31138–31150. 10.1074/jbc.m115.654855 26546678 PMC4692237

[B119] Nomura R. Kiyota A. Suzaki E. Kataoka K. Ohe Y. Miyamoto K. 2004). Human Coronavirus 229E Binds to CD13 in Rafts and Enters the Cell through Caveolae. Jvi 78(16), 8701–8708. 10.1128/jvi.78.16.8701-8708.2004 PMC479086 15280478

[B120] O’Flaherty K. Ataíde R. Zaloumis S. G. Ashley E. A. Powell R. Feng L. (2019). Contribution of Functional Antimalarial Immunity to Measures of Parasite Clearance in Therapeutic Efficacy Studies of Artemisinin Derivatives. J. Infect. Dis. 220, 1178–1187. 10.1093/infdis/jiz247 31075171 PMC6735958

[B121] O'Keefe B. R. Giomarelli B. Barnard D. L. Shenoy S. R. Chan P. K. S. McMahon J. B. (2010). Broad-Spectrum *In Vitro* Activity and *In Vivo* Efficacy of the Antiviral Protein Griffithsin against Emerging Viruses of the Family Coronaviridae. Jvi 84(5), 2511–2521. 10.1128/jvi.02322-09 PMC2820936 20032190

[B122] Oliveira A. F. Teixeira R. R. Oliveira A. S. Souza A. P. Silva M. L. Paula S. O. (2017). Potential Antivirals: Natural Products Targeting Replication Enzymes of Dengue and Chikungunya Viruses. Molecules, 22(3): 505. 10.3390/molecules22030505 PMC6155337 28327521

[B123] Packer L. Cadenas E. (2011). Lipoic Acid: Energy Metabolism and Redox Regulation of Transcription and Cell Signaling. J. Clin. Biochem. Nutr. 48(1), 26–32. 10.3164/jcbn.11-005FR 21297908 PMC3022059

[B125] Park J.-E. Li K. Barlan A. Fehr A. R. Perlman S. McCray P. B. Jr. (2016). Proteolytic Processing of Middle East Respiratory Syndrome Coronavirus Spikes Expands Virus Tropism. Proc. Natl. Acad. Sci. USA 113, 12262–12267. 10.1073/pnas.1608147113 27791014 PMC5086990

[B124] Park J.-Y. Kim J. H. Kim Y. M. Jeong H. J. Kim D. W. Park K. H. (2012). Tanshinones as Selective and Slow-Binding Inhibitors for SARS-CoV Cysteine Proteases. Bioorg. Med. Chem. 20(19), 5928–5935. 10.1016/j.bmc.2012.07.038 22884354 PMC7127169

[B126] Park J.-Y. Yuk H. J. Ryu H. W. Lim S. H. Kim K. S. Park K. H. (2017). Evaluation of Polyphenols from *Broussonetia Papyrifera* as Coronavirus Protease Inhibitors. J. Enzyme Inhib. Med. Chem. 32(1), 504–512. 10.1080/14756366.2016.1265519 28112000 PMC6010046

[B127] Peng G. Sun D. Rajashankar K. R. Qian Z. Holmes K. V. Li F. (2011). Crystal Structure of Mouse Coronavirus Receptor-Binding Domain Complexed with its Murine Receptor. Proc. Natl. Acad. Sci. 108(26), 10696–10701. 10.1073/pnas.1104306108 21670291 PMC3127895

[B128] Peng G. Xu L. Lin Y.-L. Chen L. Pasquarella J. R. Holmes K. V. (2012). Crystal Structure of Bovine Coronavirus Spike Protein Lectin Domain*. J. Biol. Chem. 287(50), 41931–41938. 10.1074/jbc.m112.418210 23091051 PMC3516740

[B129] Peng G. Yang Y. Pasquarella J. R. Xu L. Qian Z. Holmes K. V. (2017). Structural and Molecular Evidence Suggesting Coronavirus-Driven Evolution of Mouse Receptor. J. Biol. Chem. 292(6), 2174–2181. 10.1074/jbc.m116.764266 28035001 PMC5313091

[B130] Pooja M. Reddy G. J. Hema K. Dodoala S. Koganti B. (2021). Unravelling High-Affinity Binding Compounds towards Transmembrane Protease Serine 2 Enzyme in Treating SARS-CoV-2 Infection Using Molecular Modelling and Docking Studies. Eur. J. Pharmacol. 890, 173688. 10.1016/j.ejphar.2020.173688 33130280 PMC7598566

[B400] Pour P. M. Fakhri S. Asgary S. Farzaei M. H. Echeverría J. (2019). The Signaling Pathways, and Therapeutic Targets of Antiviral Agents: Focusing on the Antiviral Approaches and Clinical Perspectives of Anthocyanins in the Management of Viral Diseases. Front. Pharmacol. 10, 1207. 10.3389/fphar.2019.01207 31787892 PMC6856223

[B131] Pujhari S. Brustolin M. Macias V. M. Nissly R. H. Nomura M. Kuchipudi S. V. (2019). Heat Shock Protein 70 (Hsp70) Mediates Zika Virus Entry, Replication, and Egress from Host Cells. Emerging Microbes & Infections 8(1), 8–16. 10.1080/22221751.2018.1557988 30866755 PMC6455116

[B132] Qing Z. Chen X. Y. Martin C. (2016). *Scutellaria Baicalensis*, the Golden Herb from the Garden of Chinese Medicinal Plants. Sci. Bull. 61(18), 1391–1398. 10.1007/s11434-016-1136-5 PMC5031759 27730005

[B133] Raj V. S. Mou H. Smits S. L. Dekkers D. H. W. Müller M. A. Dijkman R. (2013). Dipeptidyl Peptidase 4 Is a Functional Receptor for the Emerging Human Coronavirus-EMC. Nature 495, 251–254. 10.1038/nature12005 23486063 PMC7095326

[B134] Rao R. V. Peel A. Logvinova A. del Rio G. Hermel E. Yokota T. 2002). Coupling Endoplasmic Reticulum Stress to the Cell Death Program: Role of the ER Chaperone GRP78. FEBS Lett. 514(2–3), 122–128. 10.1016/s0014-5793(02)02289-5 11943137 PMC3971841

[B135] Rasool N. Akhtar A. Hussain W. (2020). Insights into the Inhibitory Potential of Selective Phytochemicals against Mpro of 2019-nCoV: a Computer-Aided Study. Struct. Chem. 31, 1777–1783. 10.1007/s11224-020-01536-6 PMC7193139 32362735

[B136] Ratia K. Pegan S. Takayama J. Sleeman K. Coughlin M. Baliji S. (2008). A Noncovalent Class of Papain-like Protease/deubiquitinase Inhibitors Blocks SARS Virus Replication. Proc. Natl. Acad. Sci. 105, 16119–16124. 10.1073/pnas.0805240105 18852458 PMC2571001

[B137] Regueiro-Ren A. Swidorski J. J. Liu Z. Chen Y. Sin N. Sit S. Y. (2018). Design, Synthesis, and SAR of C-3 Benzoic Acid, C-17 Triterpenoid Derivatives. Identification of the HIV-1 Maturation Inhibitor 4-((1 R,3a S,5a R,5b R,7a R,11a S,11b R,13a R,13b R)-3a-((2-(1,1-Dioxidothiomorpholino)ethyl)amino)-5a,5b,8,8,11a-pentamethyl-1-(prop-1-en-2-yl)-2,3,3a,4,5,5a,5b,6,7,7a,8,11,11a,11b,12,13,13a,13b-octadecahydro-1 H-Cyclopenta[ A]chrysen-9-Yl)benzoic Acid (GSK3532795, BMS-955176). J. Med. Chem. 61(16), 7289–7313. 10.1021/acs.jmedchem.8b00854 30067361

[B138] Roh C. (2012). A Facile Inhibitor Screening of SARS Coronavirus N Protein Using Nanoparticle-Based RNA Oligonucleotide. Ijn 7, 2173. 10.2147/IJN.S31379 22619553 PMC3356205

[B139] Ryu Y. B. Jeong H. J. Kim J. H. Kim Y. M. Park J.-Y. Kim D. (2010). Biflavonoids from Torreya Nucifera Displaying SARS-CoV 3CLpro Inhibition. Bioorg. Med. Chem. 18, 7940–7947. 10.1016/j.bmc.2010.09.035 20934345 PMC7126309

[B140] Ryu Y. B. Park S.-J. Kim Y. M. Lee J.-Y. Seo W. D. Chang J. S. (2010). SARS-CoV 3CLpro Inhibitory Effects of Quinone-Methide Triterpenes from *Tripterygium Regelii* . Bioorg. Med. Chem. Lett. 20, 1873–1876. 10.1016/j.bmcl.2010.01.152 20167482 PMC7127101

[B141] Sayed A. A. Elfiky A. A. (2018). In Silico estrogen-like Activity and In Vivo Osteoclastogenesis Inhibitory Effect of Cicer Arietinum Extract. Cel Mol Biol (Noisy-le-grand) 64(5), 29–39. 10.14715/cmb/2018.64.5.5 29729691

[B142] Schwarz S. Sauter D. Wang K. Zhang R. Sun B. Karioti A. (2014). Kaempferol Derivatives as Antiviral Drugs against the 3a Channel Protein of Coronavirus. Planta Med. 80(02/03), 177–182. 10.1055/s-0033-1360277 24458263 PMC7171712

[B143] Sharma A. D. Kaur I. (2020). Jensenone from eucalyptus Essential Oil as a Potential Inhibitor of COVID 19 Corona Virus Infection. Res. Rev. Biotech. Biosci., 7, 59–66. 10.5281/zenodo.3748477

[B144] Sharma P. Shanavas A. (2020). Natural Derivatives with Dual Binding Potential against SARS-CoV-2 Main Protease and Human ACE2 Possess Low Oral Bioavailability: a Brief Computational Analysis. J. Biomol. Struct. Dyn. 21, 1–12. 10.1080/07391102.2020.1794970 PMC7441762 32691697

[B146] Shen L. Niu J. Wang C. Huang B. Wang W. Zhu N. (2019). High-throughput Screening and Identification of Potent Broad-spectrum Inhibitors of Coronaviruses. J. Virol. 93(12), e00023–e00019. 10.1128/jvi.00023-19 30918074 PMC6613765

[B145] Shen Y.-C. Wang L.-T. Khalil A. T. Chiang L. C. Cheng P.-W. (2005). Bioactive Pyranoxanthones from the Roots of *Calophyllum Blancoi* . Chem. Pharm. Bull. 53(2), 244–247. 10.1248/cpb.53.244 15684529

[B147] Simmons G. Zmora P. Gierer S. Heurich A. Pöhlmann S. (2013). Proteolytic Activation of the SARS-Coronavirus Spike Protein: Cutting Enzymes at the Cutting Edge of Antiviral Research. Antiviral Res. 100, 605–614. 10.1016/j.antiviral.2013.09.028 24121034 PMC3889862

[B148] Smith M. Smith J. C. (2020). Repurposing Therapeutics for COVID-19: Supercomputer-Based Docking to the SARS-CoV-2 Viral Spike Protein and Viral Spike Protein-Human ACE2 Interface, ChemRxiv 10.26434/chemrxiv.11871402.v4

[B149] Smits S. L. Gerwig G. J. van Vliet A. L. W. Lissenberg A. Briza P. Kamerling J. P. (2005). Nidovirus Sialate-O-Acetylesterases. J. Biol. Chem. 280, 6933–6941. 10.1074/jbc.m409683200 15507445 PMC8062793

[B150] Söderberg C. Giugni T. D. Zaia J. A. Larsson S. Wahlberg J. M. Möller E. (1993). CD13 (Human Aminopeptidase N) Mediates Human Cytomegalovirus Infection. J. Virol. 67 (11), 6576–6585. 10.1128/jvi.67.11.6576-6585.1993 8105105 PMC238095

[B151] Song J. Shim J. Choi H. (2011). Quercetin 7-rhamnoside Reduces Porcine Epidemic Diarrhea Virus Replication via Independent Pathway of Viral Induced Reactive Oxygen Species. Virol. J. 8, 460. 10.1186/1743-422x-8-460 21967756 PMC3200163

[B152] Song Y. H. Kim D. W. Curtis-Long M. J. Yuk H. J. Wang Y. Zhuang N. (2014). Papain-like Protease (PLpro) Inhibitory Effects of Cinnamic Amides from *Tribulus Terrestris* Fruits. Biol. Pharm. Bull. 37(6), 1021–1028. 10.1248/bpb.b14-00026 24882413

[B153] Stefaniu A. Pirvu L. Albu B. Pintilie L. (2020). Molecular Docking Study on Several Benzoic Acid Derivatives against SARS-CoV-2. Molecules 25(24), 5828. 10.3390/molecules25245828 PMC7770597 33321862

[B155] Sun P. Sun N. Yin W. Sun Y. Fan K. Guo J. (2019). Matrine Inhibits IL-1β Secretion in Primary Porcine Alveolar Macrophages through the MyD88/NF-Κb Pathway and NLRP3 Inflammasome. Vet. Res. 50(1), 53. 10.1186/s13567-019-0671-x 31300043 PMC6626430

[B154] Sun Y. Zhao Y. Yao J. Zhao L. Wu Z. Wang Y. (2015). Wogonoside Protects against Dextran Sulfate Sodium-Induced Experimental Colitis in Mice by Inhibiting NF-Κb and NLRP3 Inflammasome Activation. Biochem. Pharmacol. 94(2), 142–154. 10.1016/j.bcp.2015.02.002 25677765

[B156] Sundararajan A. Ganapathy R. Huan L. Dunlap J. R. Webby R. J. Kotwal G. J. (2010). Influenza Virus Variation in Susceptibility to Inactivation by Pomegranate Polyphenols Is Determined by Envelope Glycoproteins. Antiviral Res. 88(1), 1–9. 10.1016/j.antiviral.2010.06.014 20637243 PMC7114265

[B157] Taguchi F. Matsuyama S. (2002). Soluble Receptor Potentiates Receptor-independent Infection by Murine Coronavirus. Jvi 76, 950–958. 10.1128/jvi.76.3.950-958.2002 PMC135807 11773370

[B158] Theerawatanasirikul S. Kuo C. J. Phetcharat N. Lekcharoensuk P. (2020). *In Silico* and *In Vitro* Analysis of Small Molecules and Natural Compounds Targeting the 3CL Protease of Feline Infectious Peritonitis Virus. Antiviral Res. 174, 104697. 10.1016/j.antiviral.2019.104697 31863793 PMC7114316

[B159] Tipnis S. R. Hooper N. M. Hyde R. Karran E. Christie G. Turner A. J. (2000). A Human Homolog of Angiotensin-Converting Enzyme. J. Biol. Chem. 275(43), 33238–33243. 10.1074/jbc.m002615200 10924499

[B160] Toney J. H. Navas-Martín S. Weiss S. R. Koeller A. (2004). Sabadinine: A Potential Non-peptide Anti-severe Acute-Respiratory-Syndrome Agent Identified Using Structure-Aided Design. J. Med. Chem. 47(5), 1079–1080. 10.1021/jm034137m 14971887

[B161] Tortorici M. A. Walls A. C. Lang Y. Wang C. Li Z. Koerhuis D. (2019). Structural Basis for Human Coronavirus Attachment to Sialic Acid Receptors. Nat. Struct. Mol. Biol. 26(6), 481–489. 10.1038/s41594-019-0233-y 31160783 PMC6554059

[B162] Tresnan D. B. Levis R. Holmes K. V. (1996). Feline Aminopeptidase N Serves as a Receptor for Feline, Canine, Porcine, and Human Coronaviruses in Serogroup I. J. Virol. 70(12), 8669–8674. 10.1128/jvi.70.12.8669-8674.1996 8970993 PMC190961

[B163] Tseng C.-K. Hsu S.-P. Lin C.-K. Wu Y.-H. Lee J.-C. Young K.-C. (2017). Celastrol Inhibits Hepatitis C Virus Replication by Upregulating Heme Oxygenase-1 via the JNK MAPK/Nrf2 Pathway in Human Hepatoma Cells. Antiviral Res. 146, 191–200. 10.1016/j.antiviral.2017.09.010 28935193 PMC7113881

[B164] Tu Y.-F. Chien C.-S. Yarmishyn A. A. Lin Y.-Y. Luo Y.-H. Lin Y.-T. (2020). A Review of SARS-CoV-2 and the Ongoing Clinical Trials. Ijms 21(7), 2657. 10.3390/ijms21072657 PMC7177898 32290293

[B165] Ulasli M. Gurses S. A. Bayraktar R. Yumrutas O. Oztuzcu S. Igci M. (2014). The Effects of *Nigella Sativa* (Ns), *Anthemis Hyalina* (Ah) and Citrus Sinensis (Cs) Extracts on the Replication of Coronavirus and the Expression of TRP Genes Family. Mol. Biol. Rep. 41(3), 1703–1711. 10.1007/s11033-014-3019-7 24413991 PMC3933739

[B166] Ushio Y. Abe H. (1992). Inactivation of Measles Virus and Herpes Simplex Virus by Saikosaponin D. Planta Med. 58(02), 171–173. 10.1055/s-2006-961422 1529029

[B167] Vlasak R. Luytjes W. Spaan W. Palese P. (1988). Human and Bovine Coronaviruses Recognize Sialic Acid-Containing Receptors Similar to Those of Influenza C Viruses. Proc. Natl. Acad. Sci. 85, 4526–4529. 10.1073/pnas.85.12.4526 3380803 PMC280463

[B168] Vogel H. G. Maas J. Mayer D. (2006). Drug Discovery and Evaluation: Safety and Pharmacokinetic Assays; with 125 Tables. Springer Science & Business Media. 2006 – 889.

[B169] Walls A. C. Tortorici M. A. Bosch B.-J. Frenz B. Rottier P. J. M. DiMaio F. (2016). Cryo-electron Microscopy Structure of a Coronavirus Spike Glycoprotein Trimer. Nature 531(7592), 114–117. 10.1038/nature16988 26855426 PMC5018210

[B171] Wang G.-F. Shi L.-P. Ren Y.-D. Liu Q.-F. Liu H.-F. Zhang R.-J. (2009). Anti-hepatitis B Virus Activity of Chlorogenic Acid, Quinic Acid and Caffeic Acid In Vivo and In Vitro. Antiviral Res. 83(2), 186–190. 10.1016/j.antiviral.2009.05.002 19463857

[B172] Wang L.-J. Geng C.-A. Ma Y.-B. Huang X.-Y. Luo J. Chen H. (2012). Synthesis, Biological Evaluation and Structure-Activity Relationships of Glycyrrhetinic Acid Derivatives as Novel Anti-hepatitis B Virus Agents. Bioorg. Med. Chem. Lett. 22(10), 3473–3479. 10.1016/j.bmcl.2012.03.081 22520261

[B170] Wang S.-Q. Du Q.-S. Zhao K. Li A.-X. Wei D.-Q. Chou K.-C. (2007). Virtual Screening for Finding Natural Inhibitor against Cathepsin-L for SARS Therapy. Amino Acids 33(1), 129–135. 10.1007/s00726-006-0403-1 16998715 PMC7087620

[B173] Watanabe R. Sawicki S. G. Taguchi F. (2007). Heparan Sulfate Is a Binding Molecule but Not a Receptor for CEACAM1-independent Infection of Murine Coronavirus. Virology 366, 16–22. 10.1016/j.virol.2007.06.034 17692355 PMC7103320

[B174] Wen C.-C. Kuo Y.-H. Jan J.-T. Liang P.-H. Wang S.-Y. Liu H.-G. (2007). Specific Plant Terpenoids and Lignoids Possess Potent Antiviral Activities against Severe Acute Respiratory Syndrome Coronavirus. J. Med. Chem. 50, 4087–4095. 10.1021/jm070295s 17663539

[B175] Wen C.-C. Shyur L.-F. Jan J.-T. Liang P.-H. Kuo C.-J. Arulselvan P. (2011). Traditional Chinese Medicine Herbal Extracts of Cibotium Barometz, *Gentiana* Scabra, *Dioscorea* Batatas, *Cassia* Tora, and Taxillus Chinensis Inhibit SARS-CoV Replication. J. Traditional Complement. Med. 1(1), 41–50. 10.1016/s2225-4110(16)30055-4 PMC3942999 24716104

[B176] Weng J.-R. Lin C.-S. Lai H.-C. Lin Y.-P. Wang C.-Y. Tsai Y.-C. (2019). Antiviral Activity of *Sambucus* FormosanaNakai Ethanol Extract and Related Phenolic Acid Constituents against Human Coronavirus NL63. Virus. Res. 273, 197767 10.1016/j.virusres.2019.197767 31560964 PMC7114872

[B177] Williamson G. Kerimi A. (2020). Testing of Natural Products in Clinical Trials Targeting the SARS-CoV-2 (Covid-19) Viral Spike Protein-Angiotensin Converting Enzyme-2 (ACE2) Interaction. Biochem. Pharmacol. 178, 114123–123. 10.1016/j.bcp.2020.114123 32593613 PMC7316054

[B178] Wittemer S. M. Ploch M. Windeck T. Müller S. C. Drewelow B. Derendorf H. (2005). Bioavailability and Pharmacokinetics of Caffeoylquinic Acids and Flavonoids after Oral Administration of Artichoke Leaf Extracts in Humans. Phytomedicine 12(1), 28–38. 10.1016/j.phymed.2003.11.002 15693705

[B179] Witters L. Scherle P. Friedman S. Fridman J. Caulder E. Newton R. (2008). Synergistic Inhibition with a Dual Epidermal Growth Factor receptor/HER-2/neu Tyrosine Kinase Inhibitor and a Disintegrin and Metalloprotease Inhibitor. Cancer Res. 68(17), 7083–7089. 10.1158/0008-5472.can-08-0739 18757423

[B180] Wu C.-Y. Jan J.-T. Ma S.-H. Kuo C.-J. Juan H.-F. Cheng Y.-S. E. (2004). Small Molecules Targeting Severe Acute Respiratory Syndrome Human Coronavirus. Proc. Natl. Acad. Sci. 101(27), 10012–10017. 10.1073/pnas.0403596101 15226499 PMC454157

[B181] Wu C. Liu Y. Yang Y. Zhang P. Zhong W. Wang Y. (2020). Analysis of Therapeutic Targets for SARS-CoV-2 and Discovery of Potential Drugs by Computational Methods. Acta Pharmaceutica Sinica B, 10, 766, 788. 10.1016/j.apsb.2020.02.008 32292689 PMC7102550

[B182] Xiong X. Coombs P. J. Martin S. R. Liu J. Xiao H. McCauley J. W. (2013). Receptor Binding by a Ferret-Transmissible H5 Avian Influenza Virus. Nature 497, 392–396. 10.1038/nature12144 23615615

[B183] Xu D. Lin T.-H. Li S. Da J. Wen X.-Q. Ding J. (2012). Cryptotanshinone Suppresses Androgen Receptor-Mediated Growth in Androgen Dependent and Castration Resistant Prostate Cancer Cells. Cancer Lett. 316(1), 11–22. 10.1016/j.canlet.2011.10.006 22154085 PMC3283034

[B184] Yamagata K. Hashiguchi K. Yamamoto H. Tagami M. (2019). Dietary Apigenin Reduces Induction of LOX-1 and NLRP3 Expression, Leukocyte Adhesion, and Acetylated Low-Density Lipoprotein Uptake in Human Endothelial Cells Exposed to Trimethylamine-N-Oxide. J. Cardiovasc. Pharmacol. 74(6), 558–565. 10.1097/fjc.0000000000000747 31815868

[B192] Yang C.-W. Chang H.-Y. Lee Y.-Z. Hsu H.-Y. Lee S.-J. (2018). The Cardenolide Ouabain Suppresses Coronaviral Replication via Augmenting a Na+/K+-ATPase-dependent PI3K_PDK1 axis Signaling. Toxicol. Appl. Pharmacol. 356, 90–97. 10.1016/j.taap.2018.07.028 30053394 PMC7103114

[B191] Yang C.-W. Lee Y.-Z. Hsu H.-Y. Shih C. Chao Y.-S. Chang H.-Y. (2017). Targeting Coronaviral Replication and Cellular JAK2 Mediated Dominant NF-Κb Activation for Comprehensive and Ultimate Inhibition of Coronaviral Activity. Sci. Rep. 7(1), 4105. 10.1038/s41598-017-04203-9 28642467 PMC5481340

[B187] Yang C.-W. Lee Y.-Z. Kang I.-J. Barnard D. L. Jan J.-T. Lin D. (2010). Identification of Phenanthroindolizines and Phenanthroquinolizidines as Novel Potent Anti-coronaviral Agents for Porcine Enteropathogenic Coronavirus Transmissible Gastroenteritis Virus and Human Severe Acute Respiratory Syndrome Coronavirus. Antiviral Res. 88(2), 160–168. 10.1016/j.antiviral.2010.08.009 20727913 PMC7114283

[B185] Yang H. Yang M. Ding Y. Liu Y. Lou Z. Zhou Z. (2003). The Crystal Structures of Severe Acute Respiratory Syndrome Virus Main Protease and its Complex with an Inhibitor. Proc. Natl. Acad. Sci. 100(23), 13190–13195. 10.1073/pnas.1835675100 14585926 PMC263746

[B186] Yang Q.-Y. Tian X.-Y. Fang W.-S. (2007). Bioactive Coumarins from *Boenninghausenia Sessilicarpa* . J. Asian Nat. Prod. Res. 9(1), 59–65. 10.1080/10286020500382397 17365191

[B188] Yang Z. Wu N. Fu Y. Yang G. Wang W. Zu Y. (2010). Anti-infectious Bronchitis Virus (IBV) Activity of 1,8-cineole: Effect on Nucleocapsid (N) Protein. J. Biomol. Struct. Dyn. 28(3), 323–330. 10.1080/07391102.2010.10507362 20919748

[B189] Yang Z. Wu N. Zu Y. Fu Y. (2011). Comparative Anti-infectious Bronchitis Virus (IBV) Activity of (-)-pinene: Effect on Nucleocapsid (N) Protein. Molecules 16(2), 1044–1054. 10.3390/molecules16021044 21350392 PMC6259611

[B190] Yang Z. Wu N. Zu Y. Fu Y. (2011). Comparative Anti-infectious Bronchitis Virus (IBV) Activity of (-)-Pinene: Effect on Nucleocapsid (N) Protein. Molecules 16(2), 1044–1054. 10.3390/molecules16021044 21350392 PMC6259611

[B193] Yeager C. L. Ashmun R. A. Williams R. K. Cardellichio C. B. Shapiro L. H. Look A. T. (1992). Human Aminopeptidase N Is a Receptor for Human Coronavirus 229E. Nature 357 (6377), 420–422. 10.1038/357420a0 1350662 PMC7095410

[B194] Yeager C. L. Ashmun R. A. Williams R. K. Cardellichio C. B. Shapiro L. H. Look A. T. (1992). Human Aminopeptidase N Is a Receptor for Human Coronavirus 229E. Nature 357, 420–422. 10.1038/357420a0 1350662 PMC7095410

[B195] Yepes-Pérez A. F. Herrera-Calderon O. Sánchez-Aparicio J.-E. Tiessler-Sala L. Maréchal J.-D. Cardona-G W. (2020). Investigating Potential Inhibitory Effect of *Uncaria Tomentosa* (Cat's Claw) against the Main Protease 3CLPro of SARS-CoV-2 by Molecular Modeling. Preprints 2020, 2020060326. 10.20944/preprints202006.0326.v1 PMC7532411 33029165

[B196] Yi L. Li Z. Yuan K. Qu X. Chen J. Wang G. (2004). Small Molecules Blocking the Entry of Severe Acute Respiratory Syndrome Coronavirus into Host Cells. Jvi 78(20), 11334–11339. 10.1128/jvi.78.20.11334-11339.2004 PMC521800 15452254

[B197] Yin J. Li G. Li J. Yang Q. Ren X. (2011). In Vitroandin Vivoeffects ofHouttuynia Cordataon Infectious Bronchitis Virus. Avian Pathol. 40(5), 491–498. 10.1080/03079457.2011.605107 21848486

[B198] Yonesi M. Rezazadeh A. (2020). Plants as a Prospective Source of Natural Anti-viral Compounds and Oral Vaccines against COVID-19 Coronavirus. Preprints 2020040321:0321. 10.20944/preprints202004.0321.v1

[B199] Yook H.-S. Kim K.-H. Park J.-E. Shin H.-J. (2010). Antioxidative and Antiviral Properties of Flowering Cherry Fruits (Prunus serrulataL. var.Spontanea). Am. J. Chin. Med. 38(5), 937–948. 10.1142/s0192415x10008366 20821824

[B201] Yu J.-S. Tseng C.-K. Lin C.-K. Hsu Y.-C. Wu Y.-H. Hsieh C.-L. (2017). Celastrol Inhibits Dengue Virus Replication via Up-Regulating Type I Interferon and Downstream Interferon-Stimulated Responses. Antiviral Res. 137, 49–57. 10.1016/j.antiviral.2016.11.010 27847245 PMC7113783

[B200] Yu M.-S. Lee J. Lee J. M. Kim Y. Chin Y.-W. Jee J.-G. (2012). Identification of Myricetin and Scutellarein as Novel Chemical Inhibitors of the SARS Coronavirus Helicase, nsP13. Bioorg. Med. Chem. Lett. 22, 4049–4054. 10.1016/j.bmcl.2012.04.081 22578462 PMC7127438

[B203] Zhang B. C. Li Z. Xu W. Xiang C. H. Ma Y. F. (2018). Luteolin Alleviates NLRP3 Inflammasome Activation and Directs Macrophage Polarization in Lipopolysaccharide-Stimulated RAW264.7 Cells. Am. J. Transl. Res. 10(1), 265–273. 29423011 PMC5801364

[B202] Zhang C. H. Wang Y. F. Liu X. J. Lu J. H. Qian C. W. Wan Z. Y. (2005). Antiviral Activity of Cepharanthine against Severe Acute Respiratory Syndrome Coronavirus In Vitro. Chin. Med. J. (Engl) 118(6), 493–496. 15788131

[B204] Zhang D.-h. Wu K.-l. Zhang X. Deng S.-q. Peng B. (2020). In Silico screening of Chinese Herbal Medicines with the Potential to Directly Inhibit 2019 Novel Coronavirus. J. Integr. Med. 18(2), 152–158. 10.1016/j.joim.2020.02.005 32113846 PMC7102521

[B205] Zhou P. Yang X.-L. Wang X.-G. Hu B. Zhang L. Zhang W. (2020). A Pneumonia Outbreak Associated with a New Coronavirus of Probable Bat Origin. Nature 579(7798), 270–273. 10.1038/s41586-020-2012-7 32015507 PMC7095418

[B206] Zhu X. Liu S. Wang X. Luo Z. Shi Y. Wang D. (2018). Contribution of Porcine Aminopeptidase N to Porcine Deltacoronavirus Infection. Emerging Microbes & Infections 7(1), 1, 13. 10.1038/s41426-018-0068-3 29636467 PMC5893578

[B207] Zhuang M. Jiang H. Suzuki Y. Li X. Xiao P. Tanaka T. (2009). Procyanidins and Butanol Extract of Cinnamomi Cortex Inhibit SARS-CoV Infection. Antiviral Res. 82(1), 73–81. 10.1016/j.antiviral.2009.02.001 19428598 PMC7114128

[B208] Zuniga S. Cruz J. L. Sola I. Mateos-Gómez P. A. Palacio L. Enjuanes L. (2020). COVID-19 and the Cardiovascular System. Nat. Rev. Cardiol. 17(5), 259–260. 10.1038/s41569-020-0360-5 32139904 PMC7095524

