# Supplementary figures and images for: Anti–SARS-CoV-2 Natural Products as Potentially Therapeutic Agents
Source: Front Pharmacol. 2021 May 27;12:590509. doi: 10.3389/fphar.2021.590509 (PMC8194829; doi:10.3389/fphar.2021.590509)

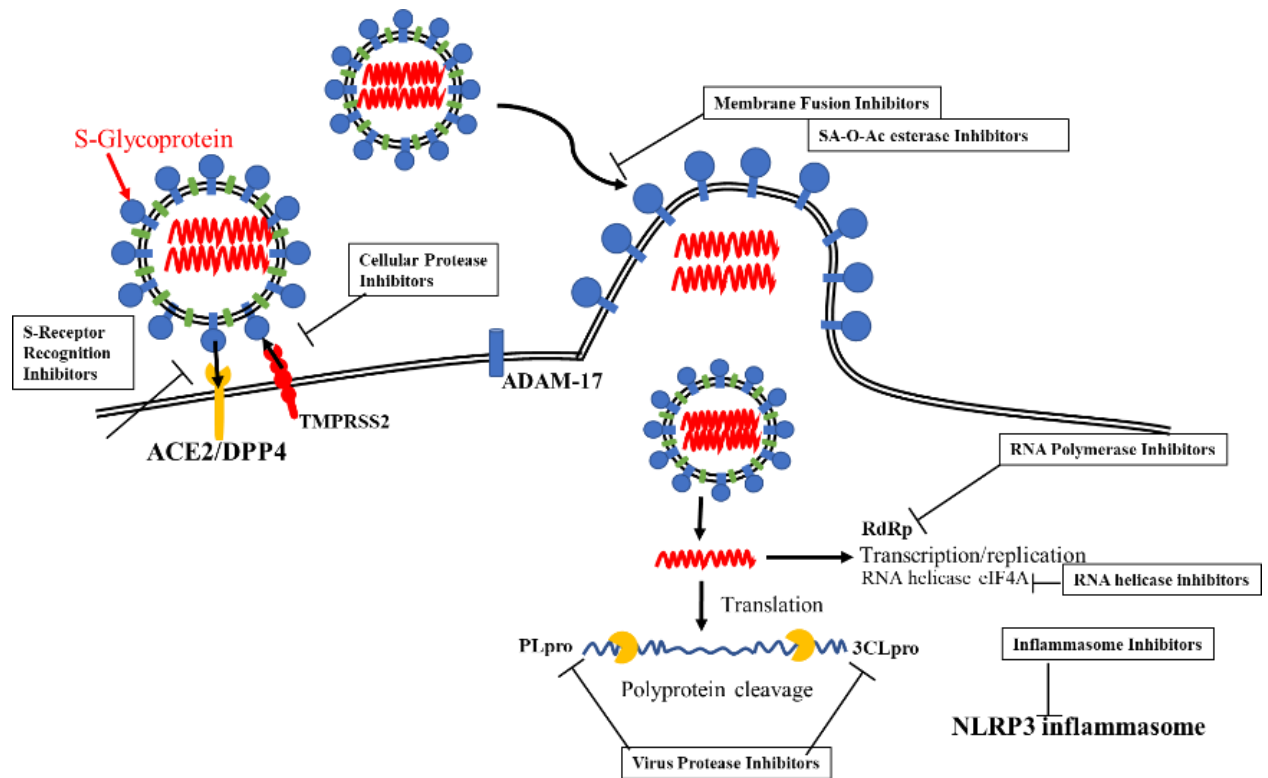

Supplement: Supplementary file 1 [file image1.pdf]
